# A Taxonomic Revision of the *Wallemia sebi* Species Complex

**DOI:** 10.1371/journal.pone.0125933

**Published:** 2015-05-27

**Authors:** Sašo Jančič, Hai D. T. Nguyen, Jens C. Frisvad, Polona Zalar, Hans-Josef Schroers, Keith A. Seifert, Nina Gunde-Cimerman

**Affiliations:** 1 Department of Biology, Biotechnical Faculty, University of Ljubljana, Ljubljana, Slovenia; 2 Department of Biology, Faculty of Science, University of Ottawa, Ottawa, Ontario, Canada; 3 Department of Systems Biology, Technical University of Denmark, Lyngby, Denmark; 4 Agricultural Institute of Slovenia, Ljubljana, Slovenia; 5 Biodiversity (Mycology), Eastern Cereal and Oilseed Research Centre, Agriculture and Agri-Food Canada, Ottawa, Ontario, Canada; 6 Centre of Excellence for Integrated Approaches in Chemistry and Biology of Proteins (CIPKeBiP), Ljubljana, Slovenia

## Abstract

*Wallemia sebi* is a xerophilic food- and air-borne fungus. The name has been used for strains that prevail in cold, temperate and tropical climates. In this study, multi-locus phylogenetic analyses, using the internal transcribed spacer (ITS) regions, DNA replication licensing factor (*MCM7*), pre-rRNA processing protein (*TSR1*), RNA polymerase II largest subunit (*RPB1*), RNA polymerase II second largest subunit (*RPB2*) and a new marker 3´-phosphoadenosine-5´-phosphatase (*HAL2*), confirmed the previous hypothesis that *W*. *sebi* presents a complex of at least four species. Here, we confirm and apply the phylogenetic analyses based species hypotheses from a companion study to guide phenotypic assessment of *W*. *sebi* like strains from a wide range of substrates, climates and continents allowed the recognition of *W*. *sebi sensu stricto* and three new species described as *W*. *mellicola*, *W*. *Canadensis*, and *W*. *tropicalis*. The species differ in their conidial size, xerotolerance, halotolerance, chaotolerance, growth temperature regimes, extracellular enzyme activity profiles, and secondary metabolite patterns. A key to all currently accepted *Wallemia* species is provided that allow their identification on the basis of physiological, micromorphological and culture characters.

## Introduction

The fungal genus *Wallemia* is based on *W*. *ichthyophaga*, which remained the only recognized *Wallemia* species until von Arx [1] recognized *Sporendonema sebi* as congeneric. Before the application of nucleic acid sequencing to fungal taxonomy, Moore [2] noted the possible presence of dolipore septa in *W*. *sebi* speculating that *Wallemia* was a basidiomycete. This was confirmed when Zalar et al. [3] applied 18S rDNA sequencing to support the establishment of the higher taxa Wallemiomycetes and Wallemiales. Wallemiomycetes then remained monogeneric until Nguyen et al. [4] added two genera, *Geminibasidium* and *Basidioascus*, in a new order Geminibasidiales, which was tentatively classified in Wallemiomycetes, although relatively distant from *Wallemia*. Zalar et al. [3] clarified species concepts within *Wallemia*, removing *W*. *ichthyophaga* from the synonymy with *W*. *sebi* proposed by von Arx [1], and demonstrating that *Torula epizoa* var. *muriae* J.J. Kickx should be recognized as a distinct *Wallemia* species, which they called *W*. *muriae* [3].

*Wallemia sebi* is the most frequently isolated and best-studied species of the genus. It is commonly involved in the spoilage of food with low water activity (a_w_) [3,5] and its air-disseminated spores [6–9] were associated with allergies, bronchial asthma and farmer’s lung disease [10–15]. Recently, Desroches et al. [16] demonstrated that human antibodies react to compounds produced by *W*. *sebi* spores. Metabolites present in spore and mycelial fragments caused inflammation and affected lung biology in an *in vivo* mouse model [17]. *Wallemia sebi* produces the metabolites walleminol, walleminone [18], azasteroid UCA1064-B [19], and the highly toxic wallimidione [16]. Walleminol is found in food contaminated by *W*. *sebi* [20], has an LD50 of 40 μg/ml for brine shrimp and a minimum inhibitory dose of 50 μg/ml for rat liver cells [21]. The UCA1064-B compound has weak activity against *Saccharomyces cerevisae* and Gram-positive bacteria and is cytotoxic to HeLa cells [19]. *Wallemia sebi* produces a cyclopentanopyridine alkaloid that exhibits antimicrobial activity towards *Enterobacter aerogenes* [22]. Botić et al. [23] showed that salt induces biosynthesis of active compounds in *W*. *sebi* that are hemolytic towards mammalian erythrocytes.

*Wallemia sebi* was recognized as one of the most xerophilic eukaryotes and often lives in diverse, harsh environments. It can grow in a wide range of water activities, whereas *W*. *muriae* only grows on media with slightly reduced water activities. *Wallemia ichthyophaga* is obligately halophilic and requires a_w_ lowering solutes for *in vitro* growth [3,24–26]. Previous analyses of the highly variable rDNA internal transcribed spacer (ITS) sequences suggested monophyly of all strains identified as *W*. *sebi*, but at least two subclades were observed [3]. Recently generated multi-locus DNA sequence data from five genes in a companion study revealed four strongly supported clades among strains, identified as phylogenetic species following the concept of genealogical concordance of phylogenetic species recognition [27]. Accordingly, *W*. *sebi* represents a species complex, which we refer to as the *W*. *sebi* species complex (WSSC).

Ecophysiological studies and global surveys of house dust [9,27] resulted in the isolation of ~70 new strains since 2005 that conform to the broad concept of the WSSC. The objectives of our study were to redefine *W*. *sebi sensu stricto* by focusing on its phenotypic characterization and to delimit the phylogenetic species. Building on the companion study [27], phylogenetic inferences were performed using ITS and partial protein-encoding gene sequences of the DNA replication licensing factor (*MCM*7), pre-rRNA processing protein (*TSR*1), RNA polymerase II largest subunit (*RPB*1), and RNA polymerase II second largest subunit (*RPB*2). Additionally, the protein coding gene for 3´-phosphoadenosine-5´-phosphatase (*HAL*2) is introduced here and tested as an additional marker for recognizing phylogenetic species within the WSSC. To support the phylogenetic species hypotheses proposed by the companion study, phenotypic variability within the WSSC was examined using culture and micromorphological characters, xerotolerance (tolerance to low a_w_), halotolerance (tolerance to NaCl), chaotolerance (tolerance to MgCl_2_) [28,29], growth temperature regimes, extracellular enzyme activity profiling and secondary metabolite patterns. Based on these results, we describe here 3 new species of *Wallemia* by adopting a “consilient taxonomy” approach.

In mycology and bacteriology, taxonomic studies that combine phenotypic and genotypic data are often referred to as polyphasic [30]. The awkwardness of the root “phase” for this term, which in English tends to refer to phases of matter and not the diverse attributes of living things, led Quaedvlieg et al. to suggest the “consolidated” species concept as an alternative phrase [31]. We prefer “consilient taxonomy” or the “consilient species concept”, because consilience is a central pillar in the development of the modern philosophy of experimental science. Wilson [32] used the word consilience, in a broader sense, however, as a metaphor to examine the relationship between art and science. Instead, we prefer to adopt the original intention of [33], where consilience indicated a scientific situation where “… an induction, obtained from one class of facts, coincides with an induction obtained from another different class.” This describes exactly the application of several different classes of data practiced in modern fungal taxonomy, and we advocate for the replacement of the phrase “polyphasic taxonomy” with “consilient taxonomy”. In our study, the “consilient taxonomy” is based on the convergence of multiple, independent data sets, as a means of delimiting species.

## Materials and Methods

### Sources of microorganisms

The strains studied (S1 Table) were assembled from previous surveys of fungi in the built environment and other low a_w_ habitats such as food, hypersaline solar salterns and salt lakes, and dry agricultural commodities such as hay, feed, and pollen. These were isolated using either dilution-to-extinction or classical microbiological isolation strategies [3,27,34]. Strains were also obtained from the CBS-KNAW Fungal Biodiversity Centre, Utrecht, The Netherlands [CBS]; Canadian Collection of Fungal Cultures, Agriculture and Agri-Food Canada, Ottawa, Canada [CCFC/DAOM]; Ex Culture Collection of the Department of Biology, Biotechnical Faculty, University of Ljubljana, Infrastructural Centre Mycosmo, MRIC UL, Ljubljana, Slovenia [EXF]; Mycotheque of the Catholic University of Louvain, Louvain la Neuve, Belgium [MUCL]; and University of Alberta Microfungus Collection and Herbarium, Edmonton, Canada [UAMH]).

### DNA extraction, PCR amplification, and sequencing

DNA was extracted as described previously [3,4]. Primer names and sequences are listed in S2 Table. Primers for *HAL*2 were newly designed, with the help of the online platforms OligoCalc (http://www.basic.northwestern.edu/biotools/oligocalc.html). The ITS, and partial sequences of the five protein-encoding genes *RPB*1, *RPB*2, *MCM*7, *TSR*1, and *HAL*2 [35–40] were amplified. Amplification of ITS, *RPB*1, *RPB*2, *MCM*7 and *TSR*1 was done as described in Nguyen et al. [27]. Amplification of *HAL*2 was done with 10× Dream Taq DNA polymerase (Fermentas), 1× Dream Taq Buffer (Fermentas), 0.1 mM dNTPs, 0.8 μM forward primer, and 0.8 μM reverse primer. The following PCR profile was used to amplify *HAL*2: 95°C for 3 min (initial denaturation), then 40 cycles at 95°C for 30 s (denaturation), 55°C for 30 sec (annealing), 72°C for 1 min (extension), followed by 72°C for 5 min (final extension). Amplified DNA fragments were separated by electrophoresis in 1% agarose gels in 0.5× TAE buffer, and visualized using Invitrogen SYBR Safe DNA gel staining. Purified PCR fragments were Sanger sequenced by commercial service providers (Macrogene Europe, Amsterdam, The Netherlands; or Microsynth Vienna, Austria). All newly generated sequences were deposited in GenBank (S1 Table).

### Sequence data, alignment and phylogenetic analyses

The sequences of each gene were aligned using MAFFT [41], and concatenated into a single data matrix with SeaView v4.4.2 [42]. PAUP4.10b [43] was used to determine the number of parsimony informative characters for the *HAL*2 alignment. Appropriate evolutionary models under the Akaike Information Criterion (AIC) were determined with jModelTest 2 [44] (S3 Table).

Bayesian phylogenetic inferences were calculated for each partition and for the combined (ITS + *RPB*1 + *RPB*2 + *MCM*7 + *TSR*1 + *HAL*2) sequence data set using MrBayes v. 3.2.2 [45]. Single gene analyses were run for 3.0 ×10^6^ generations. The analysis of ITS sequences also used *W*. *sebi* sequences from other studies obtained from GenBank: JX240410, JX317206, JX317199, JX436301 [46], HG764524, FJ524297 [47], FJ820490 [8], KF225873, KF225874, KF225857, KF225858, KF225864 [48], KF800096 [49], EU664486, EU329737, EU329736, EU486095 [50], GU941208, GU931736 [9], GU370753, GU370758, JF497133 [51], GU721563, GU721564, KC460839, FR718458, DQ33856, HQ997370, and HQ997371. The combined analysis ran for 1.0 ×10^7^ generations. Trees were sampled every 500 generations. The first 25% of trees were discarded as burn-in, and from the remaining trees, a 50% majority rule consensus tree was calculated. The alignments and trees were deposited in TreeBASE (http://treebase.org/treebase-web/home.html) under study number 16439.

Consensus trees were imported and visualised using FigTree v. 1.4.2 (http://tree.bio.ed.ac.uk/software/figtree/) or MEGA5 [52]. To estimate species boundaries, we applied the criteria described by Lutzoni et al. [53]. Internodes were considered strongly supported if they received posterior probabilities ≥0.95. MEGA5 [52] was used to calculate pair-wise distances (p-distances) between sequences. The four clades inferred through phylogenetic analysis are referred to as clades 1 to 4. Each sequence generated from a member of the WSSC was assigned to its respective clade number, which allowed the calculation of p-distances between and within the clades using Microsoft Excel.

### Physiological and morphological studies

The following strains were used for physiological and morphological studies: six strains of clade 1 (CBS 818.96, EXF-5860, MUCL 46253, CBS 136841, CBS 136845, CBS 196.56), six of clade 2 (CBS 633.66, EXF-5675, EXF-5677, EXF-8738, EXF-8747, EXF-8745), four of clade 3 (MUCL 15061, DAOM 226642, DAOM 242570, DAOM 242571), and three of clade 4 (EXF-8739, EXF-8744, EXF-8746). Xerotolerance, halotolerance and chaotolerance were determined on malt yeast agar (MYA; 1% malt extract, 1% yeast extract, 0.1% K_2_HPO_4_, 2% agar) [54], with a_w_ decreasing from 1.00 to 0.75 in ten steps for sucrose, seven for glycerol and NaCl, and nine for MgCl_2_. The non-ionic sucrose and glycerol were used as the controlling solutes for the determination of xerotolerance, ionic NaCl for halotolerance, and ionic MgCl_2_ for chaotolerance. The pH of each medium was adjusted to 6.5 with NaOH or HCl before adding agar.

The a_w_ of media were measured using a water activity meter (AquaLab, model Series 3 TE; Decagon Devices, Pullman, WA, USA). For inoculum, conidia were suspended in sterile saline (0.9% NaCl) with 0.05% Tween 80 and 0.05% agar. Strains were point-inoculated on media in 6-cm Petri dishes in three replicates for each treatment, and incubated at room temperature for 20 d. To determine cardinal growth temperatures, strains were grown on MYA with 40% sucrose (a_w_ = 0.96) and on MYA with 8% NaCl (a_w_ = 0.95), in three replicates for each, and incubated at 4°C, 10°C, 15°C, 20°C, 24°C, 30°C, 34°C, 37°C, and 42°C for 25 d [4].

Colony diameters for all physiological tests were read every 5 d. Mean colony diameters for strains assigned to each of the four clades were calculated. The coefficients of the linear parts of the growth curves generated with sucrose, glycerol, NaCl and MgCl_2_ represented the colony radial growth rates for the above specified members of each clade.

Colony characters were assessed in 9-cm plastic Petri dishes incubated at 24°C for 14 d in the dark, following three-point inoculation, on the following media: MEA [55]; MYA [54] (a_w_ ≈ 1.00); MYA plus 8% NaCl (a_w_ = 0.95), 16% NaCl (a_w_ = 0.88), 20% sucrose (a_w_ = 0.98), 50% sucrose (a_w_ = 0.94), 70% sucrose (a_w_ = 0.88), 20% glycerol (a_w_ = 0.95), and 40% glycerol (a_w_ = 0.86); and DG18 [56] (a_w_ = 0.953). Characters such as size, color, spreading tendency, structure, texture of colonies, exudate production and sporulation, and color of the colony reverse [3], were assessed with a stereomicroscope (Leica EZ4). Photographs were taken of the colonies using a Canon PowerShot G16 camera.

Micromorphological characters were defined for cultures grown on MYA with 50% sucrose (a_w_ = 0.94) incubated at 24°C for 7 d [3], and included descriptions of the hyphae, conidiophores, conidiogenous cells and conidia. Sporulating material was mounted in 60% lactic acid. Photographs were taken with an Olympus DP73 camera on an Olympus BX51 microscope. For each strain, dimensions of 100 conidia were measured with the image analysis software Cell.

### Extracellular enzyme activities

Tests to determine proteolytic (based on casein used as the substrate), amylolytic (soluble starch), cellulolytic (carboxymethyl cellulose), β-glucosidase (easculin), esterase (Tween 80), xylanase (xylan) and urease (urea) activities were carried out on 2% agar media without and with addition of 10%, 17% and 24% NaCl [57–60]. Conidia were suspended in saline (0.9%, 10%, 17%, 24% NaCl) from 7-d-old cultures grown at 24°C on MY50G and used for three-point inoculations. Cultures were incubated in the dark at 24°C for 14 d. Resulting colonies were photographed with a Canon PowerShot G16 camera.

### Analysis of secondary metabolites

Selected isolates of clade 1 (CBS 818.96, EXF-5860, MUCL 46253, CBS 110585, EXF-1441, EXF-5860), clade 2 (EXF-5675, EXF-5677, EXF-5918, MUCL 45613, UAMH 6689), clade 3 (MUCL 15061, DAOM 226642, DAOM 242570, DAOM 242571), and clade 4 (EXF-8739, EXF-8744, EXF-8746) were inoculated at three-points on YES (Yeast Extract Sucrose) agar, CYAS (Czapek Yeast Extract Agar) [56,61] plus 5% NaCl, and grown at 24°C for 10 d in the dark. Six colony plugs were excised from each medium, and pooled in 1.5-ml screw-cap vials. A mixture of methanol-dichloromethane-ethyl acetate (1:2:3 [v/v/v]) containing 0.5% formic acid was added (500 μl) for ultrasonic extraction for 60 min [62]. Organic phases were transferred to clean vials and evaporated to dryness during centrifugation under vacuum. Residues were re-dissolved in 500 μl methanol and filtered (0.45 μm filters; Sartorius).

One μl of the solutions was used for HPLC analyses (Chromeleon Dionex UHPLC; Dionex Ultimate 3000 RS Diode array detector) using alkylphenone retention indices and diode array UV/VIS detection from 200–600 nm [63]. Separations were run on a 2 × 100 nm Luna2 OOD-4251-BO-C_18_ column with a C_18_ pre-column, both packed with 3 μm particles. A linear gradient from 85% water, 15% acetonitrile was run to 100% acetonitrile over 20 min, then maintained at 100% acetonitrile for 5 min, at a flow rate of 0.4 ml min^-1^. Both eluents contained 0.005% trifluoroacetic acid. The alkylphenone retention index was calculated for each peak, and compounds were identified by retention times and UV/VIS spectra. All peaks were quantified by height, followed by qualitative and quantitative multivariate statistical analyses [64]. Quantitative secondary metabolite data were analyzed by principal component analysis (PCA) using UNSCRAMBLER (CAMO, Oslo, Norway), with correspondence and the unweighted pair group method with arithmetic mean cluster analysis using NT-SYS (Numerical Taxonomy and Multivariate System, version 2.10; Exeter Software, New York, USA) [65].

### Nomenclature

The electronic version of this article in Portable Document Format (PDF) in a work with an ISSN or ISBN will represent a published work according to the International Code of Nomenclature for algae, fungi, and plants [66], and hence the new names contained in the electronic publication of a PLOS ONE article are effectively published under that Code from the electronic edition alone.

In addition, the new names introduced here have been submitted to MycoBank from where they will be made available to the Global Names Index. The unique MycoBank number can be resolved and the associated information viewed through any standard web browser by appending the MycoBank numbers contained in this publication to the prefix http://www.mycobank.org/MB/. The online version of this work is archived and available from PubMed Central and LOCKSS.

### Ethics statement

In this study, no field activities were performed and no endangered and protected species were involved. All strains studied here were obtained from different culture collections and are considered publicly available for research purposes. To the best of our knowledge no specific permissions were required for sampling on the locations specified in S1 Table.

## Results

### Phylogenetic analyses

**Fig 1:**
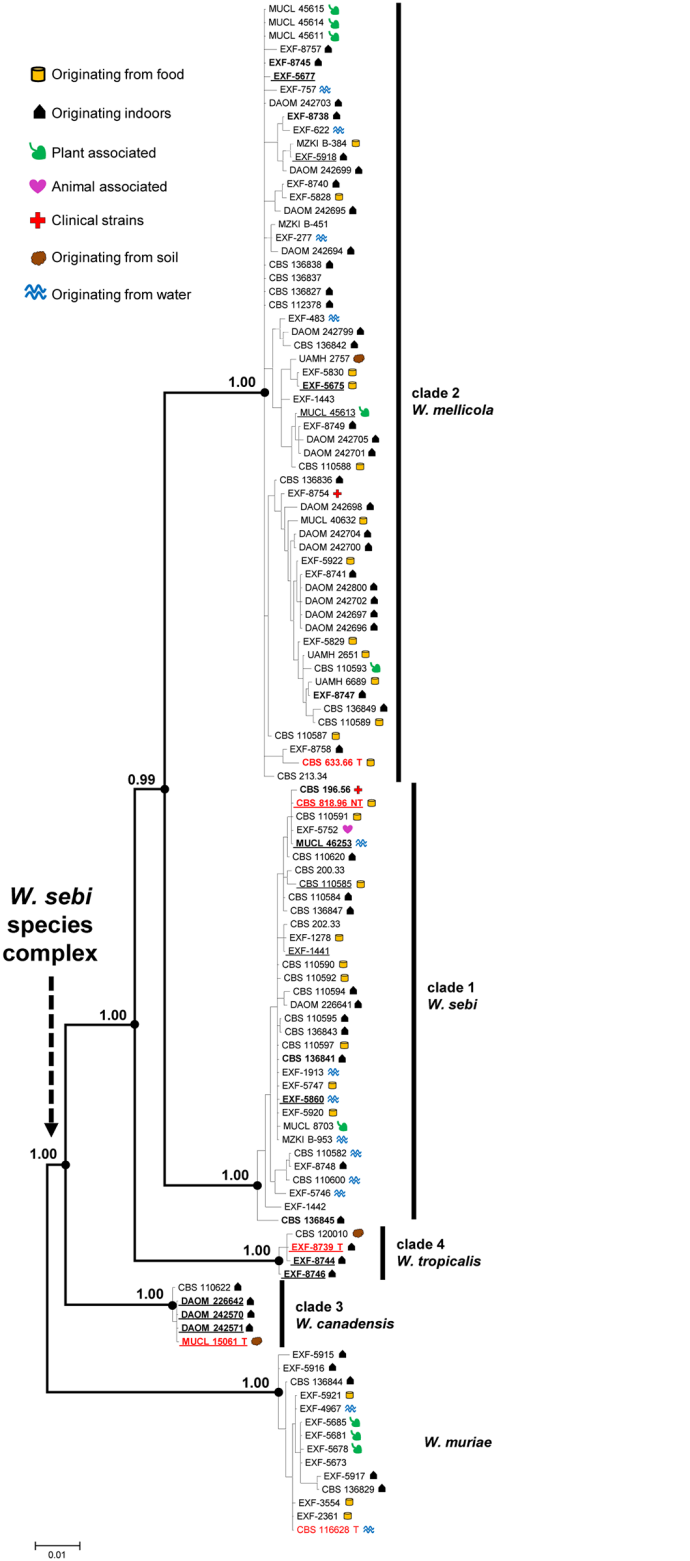
Midpoint rooted majority rule consensus tree of Bayesian MCMC sampling inferred from combined sequences. The tree from six aligned loci (ITS, *rpb*2, *rpb*1, *MCM*7, *tsr*1, *hal*2) provides a resolved structure of the WSSC. Bayesian posterior probabilities are displayed at the nodes of the tree. Labels provide information on strain number and origin. Red T, ex-type strains; red NT, ex-neotype strain; bold, strains included in physiological and morphological studies, and for the determination of extracellular enzyme activities; underlined, strains included in studies of secondary metabolites.

**Fig 2:**
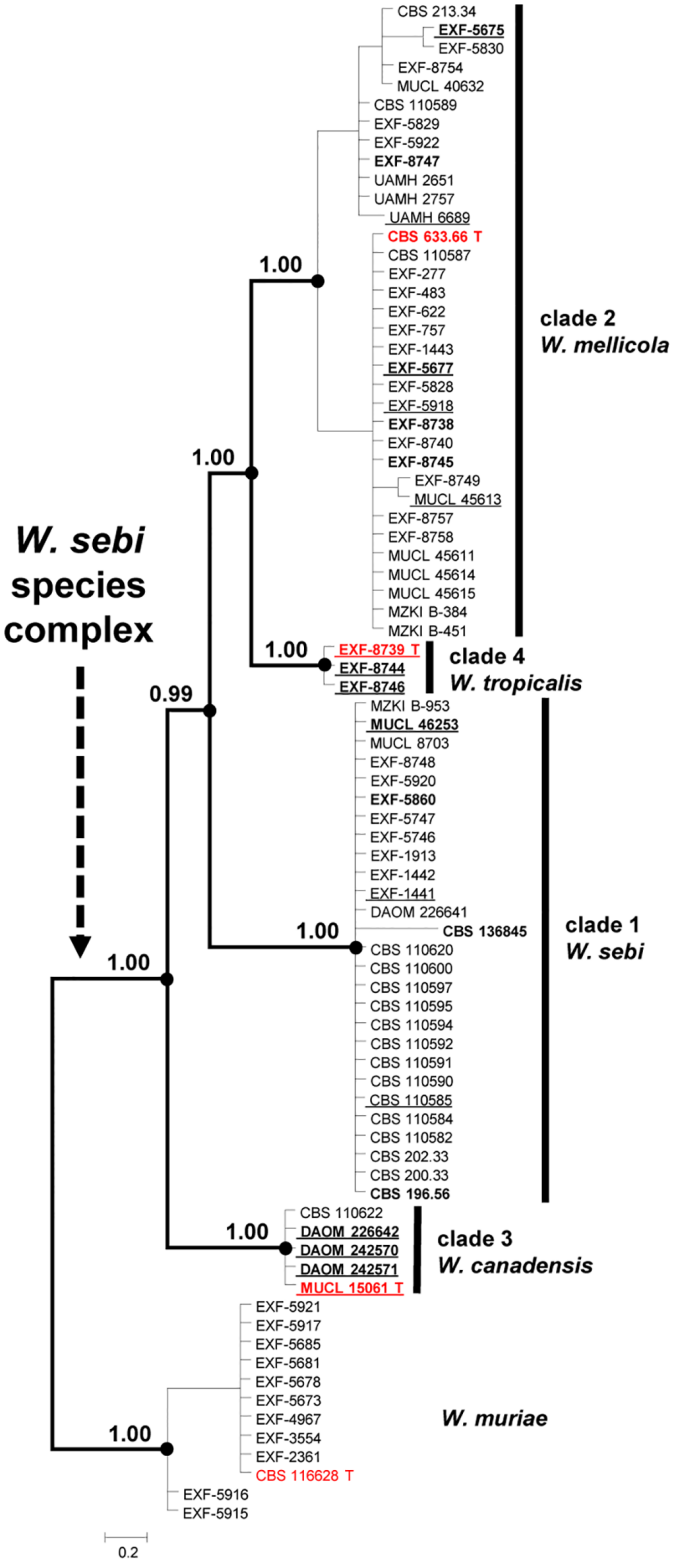
Midpoint rooted majority rule consensus tree of Bayesian MCMC sampling inferred from the *HAL*2 sequences. Bayesian posterior probabilities are displayed at the nodes of the tree. Red T, ex-type strains; red NT, ex-neotype strain; bold, strains included in physiological and morphological studies, and for the determination of extracellular enzyme activities; underlined, strains included in studies of secondary metabolites.

The ITS phylogenetic analysis divided the WSSC into two subgroups, with only the subgroup including the ex-neotype strain CBS 818.96 strongly supported. Most strains of the WSSC formed an un-resolved and weakly supported cluster (S1 Fig). Analyses of the *rpb*2, *rpb*1, *MCM*7, and *tsr*1 sequences resolved the WSSC into four groups, referred to as clades 1–4. The tree topologies based on *rpb*1, *MCM*7, and *tsr*1 (S1 File) were similar to the topology of the combined data set (Fig 1), suggesting a sister-group relationship of clades 1 and 2. Clades 1–4 also received high support in analyses of the *hal*2 sequences (Fig 2), with a supported sister group relationship for clades 2 and 4. Comparison of pair-wise distance (p-distance), alignment length, and parsimony informative characters obtained from *HAL*2 sequences are provided in S2 Fig and S3 Table. Further details of the phylogenetic analyses, determination of genetic variability of sampled loci and barcode gap analyses are described in the companion study [27].

### Physiology

All strains of the WSSC grew on media (MEA or MYA) with a_w_ ≈ 1.00. Strains from clade 2 had the highest radial growth rate (0.36 mm d^-1^; R^2^ = 0.98). Growth rates within clade 1 were 0.30 mm d^-1^ (R^2^ = 0.99), within clade 3 they were 0.27 mm d^-1^ (R^2^ = 0.99) and within clade 4 they were 0.22 mm d^-1^ (R^2^ = 0.98). Members of clades 1 and 2 grew faster than the more distantly related members of clades 3 and 4 (Table 1).

**Table 1:** Growth of strains from clades 1–4 on MEA/MYA at high salt (NaCl, MgCl_2_) concentrations and different temperatures.

Clade (n)	Growth on MEA/MYA														
	No additions	+NaCl (%)			+MgCl_2_ (%)					+8% NaCl or 40% sucrose at different temperatures (°C)					
		20	24	28	9	11	13	15	17	4	10	24	30	34	37
1 (6)	+	+	+	+	+	+	+	+	+/–	–	+	+	+ (opt.)	+	–
2 (6)	+	+	+	–	+	+	+	–	–	–	+	+	+ (opt.)	+	–
3 (4)	+	+	+	–	+	+	–	–	–	–	+	+ (opt.)	+	–	–
4 (3)	+	+	+	+/–	+	+	+	–	–	–	+	+	+ (opt.)	+	–

+, all tested strains showed visible growth;–, no tested strain showed visible growth; +/–, 50% or more tested strains showed visible growth; opt., optimal growth temperature; n, number of tested strains for each of the specified phylogenetic clades.

**Fig 3:**
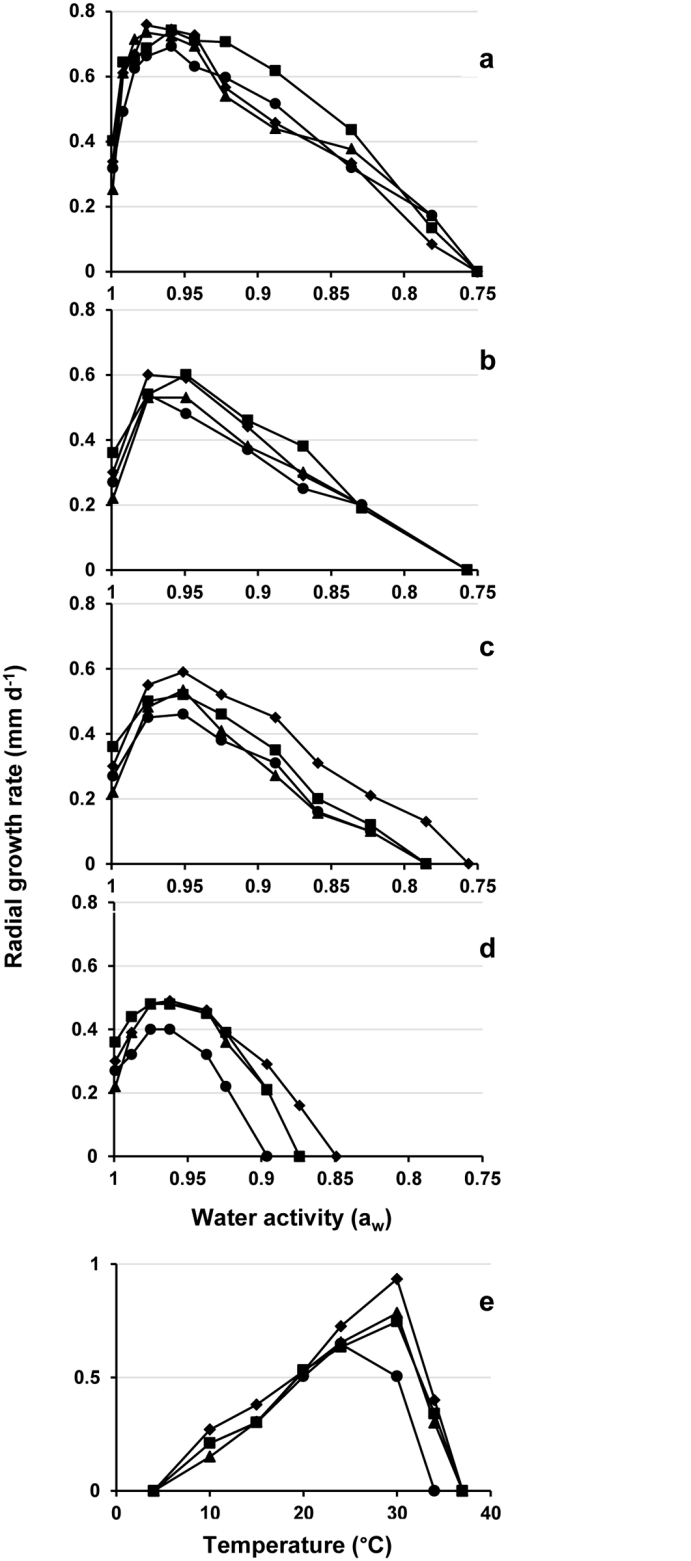
Growth parameters of the WSSC members on media with various a_w_ and solutes, and at different temperatures. (a-d) Mean colony growth rates (mm d^-1^) obtained from MYA plus various concentrations of sucrose (a), glycerol (b), NaCl (c), and MgCl_**2**_ (d). (e) Mean colony growth rates at temperatures from 4°C to 40°C on MYA with addition of 40% sucrose. Diamonds, clade 1 (*W*. *sebi*, 6 strains); squares, clade 2 (*W*. *mellicola*, 6 strains); circles, clade 3 (*W*. *canadensis*, 4 strains); triangles, clade 4 (*W*. *tropicalis*, 6 strains).

Although all strains of the WSSC grew on media without additional solutes, their growth was optimal on media with low a_w_, and they grew fastest at a_w_ 0.97 to 0.92, with all of the solutes tested. The growth rates at a_w_ 0.97 to 0.92 were 0.6–0.8 mm d^-1^ on sucrose, 0.5–0.6 mm d^-1^ on glycerol and NaCl, and 0.4–0.5 mm d^-1^ on MgCl_2_. Accordingly, the tested strains of the WSSC were xerophilic and grew best on MYA supplemented with 20%-50% sucrose. On sucrose media, the minimum a_w_ for all of the tested strains of the WSSC was *ca*. 0.78 (Fig 3 and S4 Table).

Optimal growth occurred at 8% (1.4 M) NaCl, which corresponded to a_w_ *ca*. 0.95. Accordingly, the tested strains of the WSSC are halophilic. All tested strains from clades 1 and 4 tolerated up to 28% (4.8 M) NaCl (a_w_ = 0.79), whereas the highest tolerated NaCl concentration for clades 2 and 3 was *ca*. 24% (4.1 M) NaCl (a_w_ = 0.82) (Table 1).

Strains from clade 1 tolerated up to 17% (1.8 M) MgCl_2_ (a_w_ = 0.85), strains from clades 2 and 4 tolerated up to 13% (1.4 M) MgCl_2_ (a_w_ = 0.90), and strains from clade 3 tolerated up to 11% (1.2 M) MgCl_2_ (a_w_ = 0.92) (Table 1). Accordingly, the tested strains of the WSSC are chaophilic or at least chaotolerant. Additional growth parameters of these members of clades 1–4 with different solutes and at different a_w_ are given in the S4 Table.

On MYA with 40% sucrose, the optimum growth temperature for the tested strains of clades 1, 2, and 4 was 30°C (growth minimum, 10°C; growth maximum, 34°C). Clade 3 grew best at 24°C (growth minimum, 10°C; growth maximum, 30°C). Growth rates at the optimum temperature were 0.93 mm d^-1^ (R^2^ = 0.99) for clade 1, 0.75 93 mm d^-1^ (R^2^ = 0.99) for clade 2, 0.60 93 mm d^-1^ (R^2^ = 0.99) for clade 3, and 0.80 93 mm d^-1^ (R^2^ = 0.99) for clade 4. No growth occurred at 4°C and 37°C (Fig 3e, Table 1). The cardinal temperatures obtained on MYA with the addition of 8% (a_w_ = 0.95) NaCl were similar to those on 40% sucrose (a_w_ = 0.96).

### Micromorphology

Mature conidia were generally spherical, slightly verrucose, thick-walled, and pale brown. On average, the smallest conidia were seen in clade 1 (diameter, 2.1 μm; standard deviation [SD], 0.2 μm; standard error [SE], 0.010 μm), and the largest in clade 2 (diameter, 2.6 μm; SD, 0.3 μm; SE, 0.011 μm). Intermediate values characterized clade 3 (diameter, 2.3 μm; SD, 0.2 μm; SE, 0.012 μm) and clade 4 (diameter, 2.4 μm; SD, 0.3 μm; SE, 0.015 μm). Other micromorphological characters for dimensions of hyphae, conidiophores and conidiogenous cells did not differ among clades 1–4.

### Extracellular enzyme activities

Proteolytic, amylolytic, cellulolytic and xylanase activity were not detected in any WSSC strains at any salinity. However, β-glucosidase, esterase and urease activities were seen for members of all four clades, with or without 10% NaCl. No enzymatic activities were detected at 24% NaCl, although β-glucosidase activity was still detected at 17% NaCl for strains of clades 1 and 2. Strains from clade 1 showed strong urease activities only under non-saline conditions (0% NaCl), although they grew in 10% and 17% NaCl. Strains from clade 3 grew and showed urease activity only in 10% NaCl; similarly, clade 4 grew and showed β-glucosidase activity only in 10% NaCl (Table 2).

**Table 2:** Extracellular enzyme activities of strains from clades 1–4 grown at different NaCl concentrations at 24°C.

Clade (n)	β-Glucosidase activity, according to NaCl (%)				Esterase activity, according to NaCl (%)				Urease activity, according to NaCl (%)			
	0	10	17	24	0	10	17	24	0	10	17	24
1 (6)	+/–	+	+	–	+	+/–	–	–	+	–	–	–
2 (6)	–	+	+/–	–	+	–/+	–	–	+	+	–	–
3 (4)	+	+	–	–	+	–	–	–	–	+	–	–
4 (3)	–	+	–	–	–/+	–	–	–	+	+	–	–

+, all tested strains showed measurable enzyme activity;–, no tested strain showed measurable enzyme activity; +/–, 50% or more tested strains showed measurable enzyme activity;–/+, 50% or less showed measurable enzyme activity; n, number of tested strains for each of the specified phylogenetic clades.

### Secondary metabolites

Forty-six compounds were detected from strains of the WSSC grown on YES agar and CYAS. The provisional identification of these compounds, their retention times, and their characteristic UV/VIS spectra are listed in S5 Table. Clade 2 members produced 28 different compounds, while 23 secondary metabolites were detected for clade 1, and 16 for clade 3. No compounds were detected for clade 4 members grown on YES agar and CYAS. Strains of clades 1 and 2 that are closely related phylogenetically produced similar metabolites, with 20 of the metabolites common to both clades. None of the metabolites detected for clade 3 members were detected for clades 1 and 2. To determine potential groupings suggested by these secondary metabolites, their quantitative amounts detected by HPLC were subjected to PCA, correspondence and cluster analyses. These relative quantitative amounts varied from 3 to 700 absorbance units (mAU) among the isolates examined. Strains of clade 3 were strongly discriminated in the PCA (S3 Fig), correspondence and cluster analyses. PCA also showed that the metabolite profiles from YES agar and CYAS were clearly different, and that NaCl had a strong impact on the production of these secondary metabolites.

### Taxonomy

By applying the genealogical concordance phylogenetic species recognition concept [67], we confirmed the results of the companion study [27] and that the WSSC consists of four phylogenetic species. Secondary metabolite profiles support the phylogenetic inference that clade 1 and clade 2 are closely related, and that clade 3 presents a taxon clearly separated from clade 1 and 2 members. Clade 1 members, including the ex-neotype isolate of *W*. *sebi*, comprise the most halotolerant and chaotolerant taxon and are physiologically distinguishable from clades 2, 3 and 4. Clade 3 is clearly distinct from the others by optimal and maximal growth temperatures, halotolerance and chaotolerance, and its secondary metabolite profile. These data thus allow a consilient delineation of species within the WSSC and the description of three distinct *Wallemia* species that are here newly named *W*. *mellicola*, *W*. *tropicalis* and *W*. *canadensis* (see summary of characters in Table 3).

**Table 3:** Summary of the characteristics of the species studied here.

Character	Detail	*W*. *sebi*	*W*. *mellicola*	*W*. *canadensis*	*W*. *tropicalis*
Classification	WSSC Clade number	clade 1	clade 2	clade 3	clade 4
	MycoBank number	325537	810412	810413	810414
	Type strain	CBS 818.96	CBS 633.66	MUCL 15061	EXF-8739
	Number of tested strains	6	6	4	3
Growth rate	MEA and MYA	0.3	0.36	0.27	0.22
(mm d^-1^)	MY50G or MYA + 50% sucrose	0.73	0.71	0.62	0.69
	MYA + 70% sucrose	0.46	0.62	0.51	0.51
	DG18 or MYA + 20% glycerol	0.59	0.6	0.48	0.53
	MEA or MYA + 16% NaCl	0.45	0.35	0.31	0.27
Conidial size	Range	1.5–2.5	2.5–3.0	2.0–2.5	2.0–3.0
(μm)	Mean ±standard deviation	2.1 ±0.2	2.6 ±0.3	2.3 ±0.2	2.4 ±0.3
Cardinal temperature	Minimum	10	10	10	10
(°C)	Optimal	30	30	24	30
	Maximum	34	34	30	34
Xerotolerance	Minimum	1	1	1	1
(a_w_)	Optimal	0.97–0.92	0.97–0.92	0.97–0.95	0.97–0.92
	Maximum	0.78	0.78	0.78	0.78
Halotolerance	Minimum	0	0	0	0
(NaCl, %)	Optimal	4–12	4–12	4–8	4–8
	Maximum	28	24	24	28
Chaotolerance	Minimum	0	0	0	0
(MgCl_2_, %)	Optimal	6–9	4–6	4–6	4–6
	Maximum	17	13	11	13

**Fig 4:**
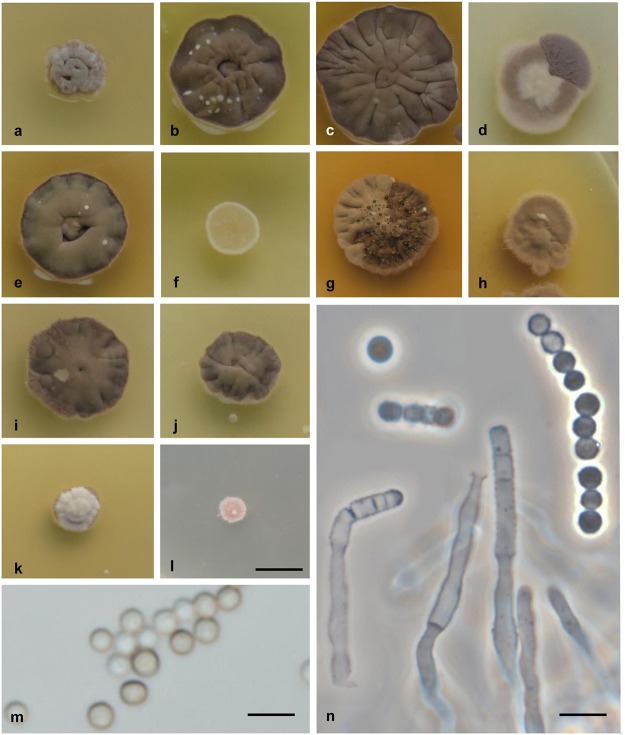
Culture and micromorphological characters for *W*. *sebi* (clade 1; ex-neotype strain CBS 818.96). (a-l) Colony surface grown on MYA with no additives (a), and MYA with the addition of 20% (b), 50% (c) and 70% (d) sucrose, 20% (e) and 40% (f) glycerol, 4% (g) and 13% (h) MgCl_**2**_, and 8% (i), 16% (j), 24% (k) and 28% (l) NaCl. (m) Conidia from MYA plus 50% sucrose. (n) Conidiogenous cell producing conidia in chains. Colonies were incubated for 14 d at 24°C. Scale bars: 5 mm (l) (applies also for a-k), 5 μm (m, n).

***Wallemia sebi* (Fr.) von Arx, The Genera of Fungi Sporulating in Pure Culture: 166. 1970.** (Fig 4).

#### Colony characteristics

Colonies on MEA and MYA: 4–6 mm diam. after 2 weeks; growth rate, 0.30 mm d^-1^; cerebriform, extending deeply into the agar; compact, pale brown or almost white, without sporulation and exudates; margin pale brown or the same color as the colony, and irregular; gray reverse. Colonies on MY50G or MYA with addition of 50% sucrose: 10–13 mm diam. after 2 weeks; growth rate, 0.73 mm d^-1^; cerebriform, spreading into the agar; compact, greenish and yellowish brown centrally, dark brown marginally, and powdery because of strong sporulation, without exudates; margin brown, and irregular; dark gray reverse. Colonies on MYA with addition of 70% sucrose: 6–8 mm diam. after 2 weeks; growth rate, 0.46 mm d^-1^; flat, pale brown, sporulation moderate, without exudates; margin white or pale brown, and regular; gray reverse. Colonies on DG18 or MYA with addition of 20% glycerol: 8–10 mm diam. after 2 weeks; growth rate, 0.59 mm d^-1^; punctiform to somewhat cerebriform, extending deeply into the agar; compact, dark brown with brighter central part, sporulation weak, without exudates; margin darker than colony, and regular; dark gray reverse. Colonies on MEA or MYA with addition of 16% NaCl: 6–8 mm diam. after 2 weeks, growth rate, 0.35 mm d^-1^; punctiform, extending deeply into the agar; compact, pale brown or white, sporulation weak, without exudates; margin pale brown or the same color as the colony, and irregular; gray reverse.

#### Conidial size

1.5–2.5 μm diam. (mean ±standard deviation, 2.1 ±0.2 μm). For description of hyphae, conidiophores and conidiogenous cells see [3].

#### Cardinal temperatures

Minimum 10°C, optimum 30°C, maximum 34°C. No growth at 4°C or 37°C.

#### Physiology

Growth at a_w_ ≈1.00 (0% NaCl, 0% MgCl_2_) positive; optimum at a_w_ = 0.97 to 0.92 (4%-12% NaCl, 6%-9% MgCl_2_); maximum at a_w_ = 0.78 (28% NaCl, 17% MgCl_2_).

#### Extracellular enzyme activities

β-glucosidase at 0% to 17% NaCl; esterase at 0% to 10% NaCl; urease at 0% NaCl.

#### Habitat

Sea salt, hypersaline water in solar salterns and salt lakes, hay; air and dust in indoor environments (house, office, archives); occasionally in pond water, mineral water, and seeds (sunflower, wheat, rye, barley, maize).

#### Distribution

Worldwide (Africa, Asia, Europe, North America).

#### Human and animal pathogenicity

Chronic ulcerative skin lesion in man (one case reported, Groningen, The Netherlands); fatal livestock toxicosis associated with hay contaminated with *W*. *sebi* (one case reported, Berkshire, UK) (*fide* [3]).

#### Typification

Sweden, dried MEA culture of CBS 818.96, originating from sunflower seed, collected and isolated by M. Olsen in 1986, neotype designated by Zalar et al. [3], deposited in CBS herbarium. Living ex-type strain: CBS 818.96 = EXF-958.

#### Cultures examined

See S1 Table.

#### Diagnostic characters

Growth positive on media without additional solutes, such as MYA or MEA, conidia 1.5–2.5 μm diam., halotolerance up to 28% NaCl, chaotolerance up to 17% MgCl_2_, maximum growth temperature 34°C, β-glucosidase activity up to 17% NaCl, no urease activity at 10% NaCl, occurrence worldwide.

#### Note

Zalar et al. mentioned *Torula minuta* Saito as a possible synonym of *W*. *sebi* because of its halotolerance, but this species is now commonly referred to as *Rhodotorula minuta* (Saito) F.C. Harrison [68]. *Wallemia sebi* was referred to as *W*. *sebi* clade 1 in Nguyen et al. [27].

**Fig 5:**
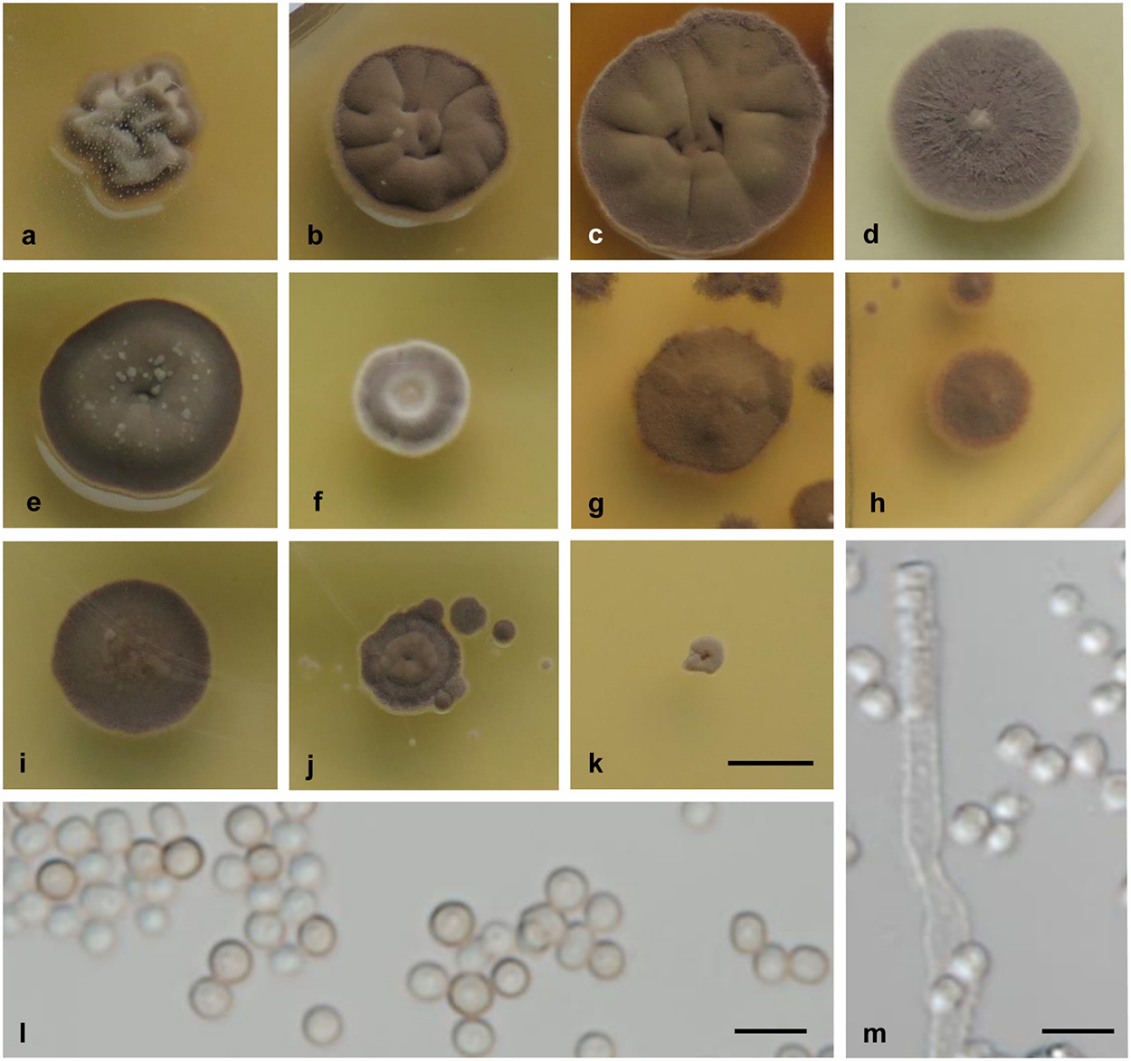
Culture and micromorphological characters for *W*. *mellicola* (clade 2; ex-type strain CBS 633.66). (a-k) Colony surface grown on MYA without additions (a), and MYA with the addition of 20% (b), 50% (c) and 70% (d) sucrose, 20% (e) and 40% (f) glycerol, 4% (g) and 13% (h) MgCl_**2**_, and 8% (i), 16% (j) and 24% (k) NaCl. (l, m) Conidia (l) and conidiophore and conidiogenous cell (m) from MYA plus 50% sucrose. Colonies were incubated for 14 d at 24°C. Scale bars: 5 mm (k) (applies also for a-j), 5 μm (l, m).

***Wallemia mellicola* Jancic, Nguyen, Seifert & Gunde-Cimerman, sp. nov.** MycoBank MB: 810412 (Fig 5).

#### Etymology

Latin *mel*, meaning honey and-*colo*, to reside, referring to the habitat of the type strain.

#### Colony characters

Colonies on MEA and MYA: 5-7mm diam. after 2 weeks; growth rate, 0.36 mm d^-1^; cerebriform, extending deeply into the agar; compact, pale brown or brown, without sporulation and exudates; margin brown or darker than the colony, irregular; dark gray reverse. Colonies on MY50G or MYA with addition of 50% sucrose: 9–12 mm diam. after 2 weeks; growth rate, 0.71 mm d^-1^; cerebriform in the central part, flat marginally, extending deeply into the agar; compact, greenish brown in the central part, brown marginally, powdery because of strong sporulation, without exudates; margin brown, and irregular; dark gray reverse. Colonies on MYA with addition of 70% sucrose: 8–10 mm diam. after 2 weeks; growth rate, 0.62 mm d^-1^; flat, pale brown, sporulation strong, without exudates; margin white or pale brown, and regular; gray reverse. Colonies on DG18 or MYA with addition of 20% glycerol: 7–11 mm diam. after 2 weeks; growth rate, 0.60 mm d^-1^; punctiform, extending deeply into the agar; very compact, dark brown with brighter central part, sporulation weak, without exudates; margin and colony the same color, and regular; dark gray reverse. Colonies on MEA or MYA with the addition of 16% NaCl: 5–7 mm diam. after 2 weeks; growth rate, 0.35 mm d^-1^; cerebriform, spreading into the agar; compact, pale brown, sporulation weak, without exudates; margin and colony the same color, and irregular; gray reverse.

#### Conidial size

2.5–3.0 μm diam. (mean ±standard deviation, 2.6 ±0.3 μm).

#### Cardinal temperatures

Minimum 10°C, optimum 30°C, maximum 34°C. No growth at 4°C or 37°C.

#### Physiology

Growth at a_w_ ≈1.00 (0% NaCl, 0% MgCl_2_) positive; optimum at a_w_ = 0.97 to 0.92 (4%-12% NaCl, 4%-6% MgCl_2_); maximum at a_w_ = 0.78 (24% NaCl, 13% MgCl_2_).

#### Extracellular enzyme activities

β-glucosidase at 10% to 17% NaCl; esterase at 0% to 10% NaCl; urease at 0% to 10% NaCl.

#### Habitat

Soil, forest plants, hypersaline water of solar salterns, salty food products (peanuts, dried fish), sugary food products (date honey, cakes, jam, maple syrup, chocolate), dried food products (bread, coconut pulp), seeds, straw, pollen; air, dust and surfaces in indoor environments.

#### Distribution

Worldwide (Asia, Europe, North America, Middle America, South America, Micronesia).

#### Human and animal pathogenicity

Subcutaneous lesion (phaeohyphomycosis) on foot in an immunocompetent human patient (Varanasi, Uttar Pradesh, India) [69].

#### Typification

Unknown origin (possibly Israel), from date honey, 63.5% total soluble solids, collected and isolated by R. B. Kenneth in 1966, holotype, designated here, CBS herbarium H-13315 (originally identified as *W*. *sebi*). Living ex-type strain: CBS 633.66 = ATCC MYA-4683 = EXF-956.

#### Cultures examined

See S1 Table.

#### Diagnostic characters

Growth positive on media without additional solutes, such as MYA or MEA, conidia 2.5–3.0 μm diam., halotolerance up to 24% NaCl, chaotolerance up to 13% MgCl_2_, growth positive at 34°C, no β-glucosidase activity without NaCl, urease activity up to 10% NaCl, occurrence worldwide.

#### Note

The genome of the strain CBS 633.66 was sequenced (under the name *W*. *sebi*) by the Joint Genome Institute at the Department of Energy, USA [70]. Wheeler et al. [54] studied FRR 3051 (= CBS 110589, EXF-1277) isolated from the dried salted fish *Ophiocephalus striatus* and concluded that this strain grows optimally at 25°C. Gock et al. [71] observed that conidial germination but no growth occurred in FRR 3051 even at 37°C. This species was referred to as *W*. *sebi* clade 2 by Nguyen et al. [27].

**Fig 6:**
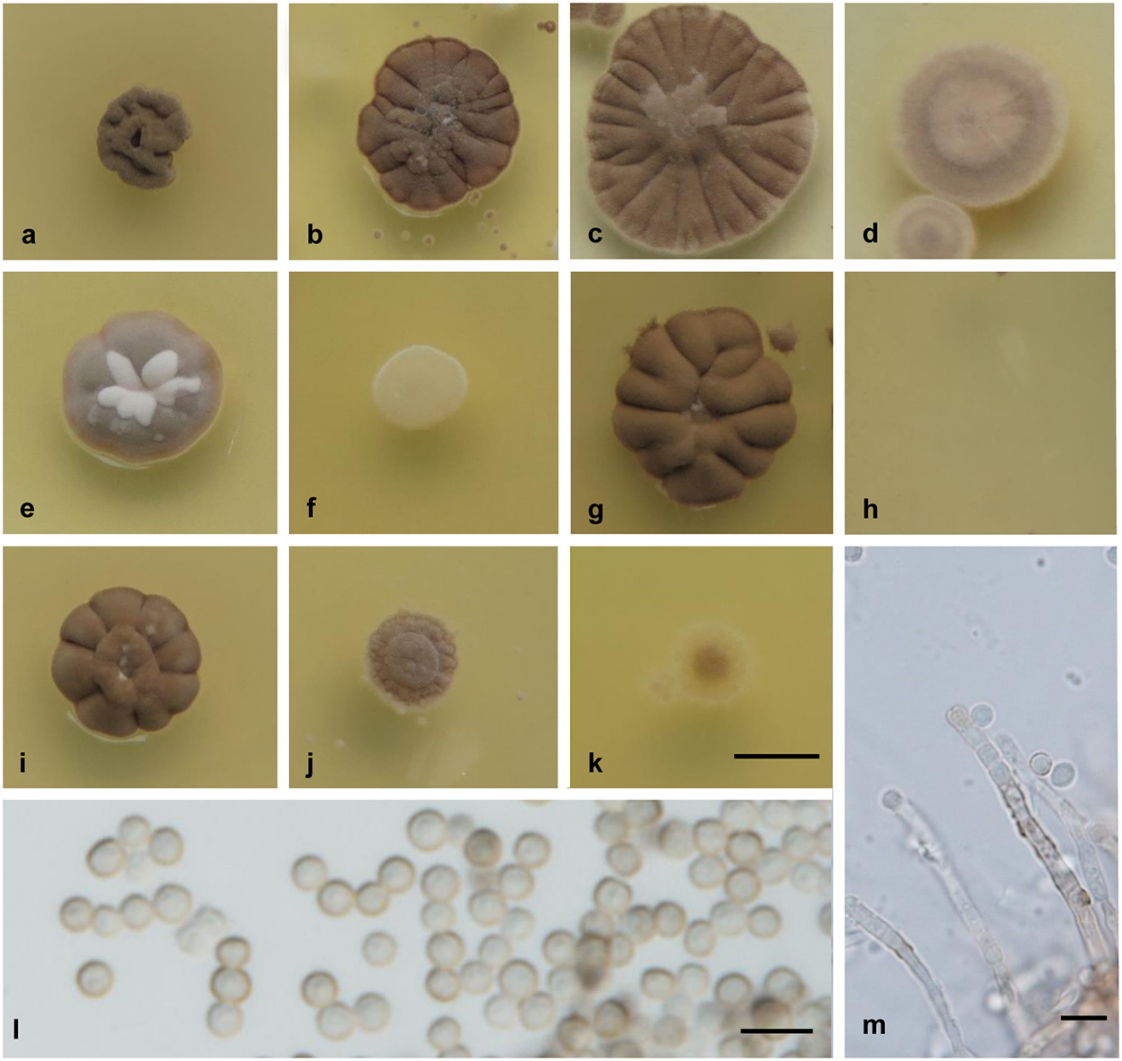
Culture and micromorphological characters for *W*. *canadensis* (clade 3; ex-type strain MUCL 15061). (a-k) Colony surface grown on MYA without additions (a), and MYA with the addition of 20% (b), 50% (c) and 70% (d) sucrose, 20% (e) and 40% (f) of glycerol, 4% (g) MgCl_**2**_ (no growth on MYA with addition of 13% (h) MgCl_**2**_), and 8% (i), 16% (j) and 24% (k) NaCl. (l, m) Conidia (l) and conidiogenous cell (m) from MYA plus 50% sucrose. Colonies were incubated for 14 d at 24°C. Scale bars: 5 mm (k) (applies also for a-j), 5 μm (l, m).

***Wallemia canadensis* Jancic, Nguyen, Seifert & Gunde-Cimerman, sp. nov.** MycoBank: MB 810413 (Fig 6).

#### Etymology

The epithet *canadensis* refers to Canada, where four of the currently five known strains were isolated.

#### Colony characters

Colonies on MEA and MYA: 4–5 mm diam. after 2 weeks; growth rate, 0.27 mm d^-1^; cerebriform, extending deeply into the agar; compact, brown, sporulation weak, without exudates; margin the same color as the colony, and irregular; gray reverse. Colonies on MY50G or MYA with addition of 50% sucrose: 7–12 mm diam. after 2 weeks; growth rate, 0.62 mm d^-1^; cerebriform, extending deeply into the agar; compact, walnut or pale brown, powdery because of strong sporulation, without exudates; margin white or the same color as the colony, and irregular; dark gray reverse. Colonies on MYA with addition of 70% sucrose: 7–10 mm diam. after 2 weeks; growth rate, 0.51 mm d^-1^; flat in concentric circles, pale brown, sporulation weak, without exudates; margin white or pale brown, and irregular; gray reverse. Colonies on DG18 or MYA with addition of 20% glycerol: 6–8 mm diam. after 2 weeks; growth rate, 0.48 mm d^-1^; slightly cerebriform, extending deeply into the agar; compact, pale brown with white central part, sporulation weak, without exudates; margin pale brown, and regular; gray reverse. Colonies on MEA or MYA with the addition of 16% NaCl: 4–5 mm diam. after 2 weeks; growth rate, 0.31 mm d^-1^; punctiform, extending deeply into the agar; compact, pale brown or white, sporulation weak, without exudates; margin the same color as the colony, and irregular; dark gray reverse.

#### Conidial size

2.0–2.5 μm diam. (mean ±standard deviation, 2.3 ±0.2 μm).

#### Cardinal temperatures

Minimum 10°C, optimum 24°C, maximum 30°C. No growth at 4°C, 34°C or 37°C.

#### Physiology

Growth at a_w_ ≈1.00 (0% NaCl, 0% MgCl_2_) positive; optimum at a_w_ = 0.97 to 0.95 (4%-8% NaCl, 4%-6% MgCl_2_); maximum at a_w_ = 0.78 (24% NaCl, 11% MgCl_2_).

#### Extracellular enzyme activities

β-glucosidase at 0% to 10% NaCl; esterase at 0% NaCl; urease at 10% NaCl.

#### Habitat

Cedar swamp, catwalk in silos; indoor dust and air.

#### Distribution

Temperate and cold climates (Canada, UK, Finland).

#### Human and animal pathogenicity

Unknown.

#### Typification

Canada (Ontario, Puslinch), from peat soil in a cedar swamp, collected and isolated by G. C. Bhatt in May 1964, holotype, designated here, herbarium CBS H-22007, consisting of a freeze-dried living but metabolically inactivated deposit of spores and mycelium from MY50G. Living ex-type strain: MUCL 15061 = UAMH 2817 = EXF-6149.

#### Cultures examined

See S1 Table.

#### Diagnostic characters

Growth on media without additional solutes such as MYA or MEA, conidia 2.0–2.5 μm, halotolerance up to 24% NaCl, chaotolerance up to 11% MgCl_2_, no growth at 34°C, no urease activity without NaCl, no esterase activity at 10% NaCl, occurrence in temperate and cold climates.

#### Note

Strain BF036 was reported as *W*. *sebi*, isolated from house dust of moisture-damaged buildings before and after renovations in Finland (GenBank number FR718458), but ITS sequences are nearly identical with the ex-type ITS sequence of *W*. *canadensis*. This species was referred to as *W*. *sebi* clade 3 by Desroches et al. [16] and Nguyen et al. [27].

**Fig 7:**
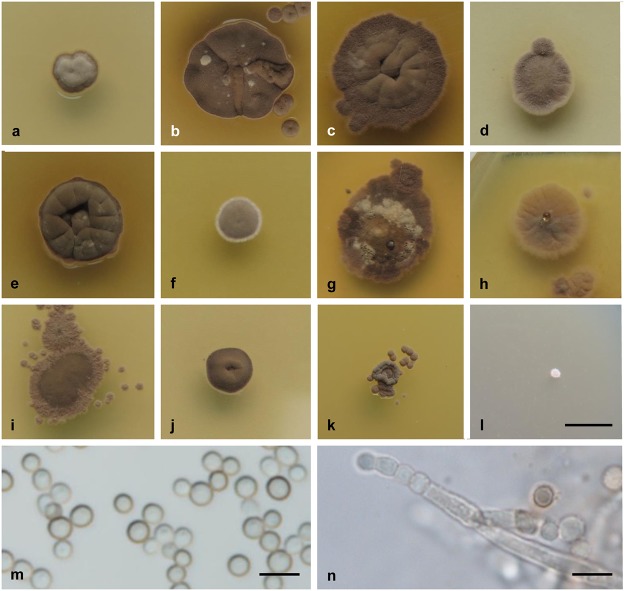
Culture and micromorphological characteristics for *W*. *tropicalis* (clade 4; ex-type strain EXF-8739). (a-l) Colony surface grown on MYA with no additions (a), and MYA with the addition of 20% (b), 50% (c) and 70% (d) sucrose, 20% (e) and 40% (f) glycerol, 4% (g) and 13% (h) MgCl_**2**_, and 8% (i), 16% (j), 24% (k) and 28% (l) NaCl. (m, n) Conidia (m) and conidiogenous cell (n) from MYA plus 50% sucrose. Colonies were incubated for 14 d at 24°C. Scale bars: 5 mm (l) (applies also for a-k), 5 μm (m, n).

***Wallemia tropicalis* Jancic, Nguyen, Seifert & Gunde-Cimerman, sp. nov.** MycoBank: MB 810414 (Fig 7).

#### Etymology

The epithet *tropicalis* refers to the tropical (subtropical) origin of the species.

#### Colony characters

Colonies on MEA and MYA: 3–5 mm diam. after 2 weeks; growth rate, 0.22 mm d^-1^; punctiform, extending deeply into the agar; compact, pale brown or almost gray, without sporulation and exudates; margin brown or the same color as the colony, and irregular; dark gray reverse. Colonies on MY50G or MYA with addition of 50% sucrose: 9–11 mm diam. after 2 weeks; growth rate, 0.69 mm d^-1^; cerebriform in the central part and flat marginally, extending deeply into the agar; compact, brown to dark brown, powdery because of strong sporulation, without exudates; margin the same color as the colony, and irregular; gray reverse. Colonies on MYA with addition of 70% sucrose: 6–7 mm diam. after 2 weeks; growth rate, 0.51 mm d^-1^; flat, pale brown, weak sporulation, without exudates; margin white or pale brown, and irregular; gray reverse. Colonies on DG18 or MYA with addition of 20% glycerol: 7–9 mm diam. after 2 weeks; growth rate, 0.53 mm d^-1^; slightly cerebriform, extending deeply into the agar; compact, dark brown, sporulation weak, without exudates; margin the same color as the colony, and regular; gray reverse. Colonies on MEA or MYA with the addition of 16% NaCl: 4–5 mm diam. after 2 weeks; growth rate, 0.27 mm d^-1^; cerebriform, spreading into the agar; compact, pale brown, sporulation weak, without exudates; margin the same color as the colony, and irregular; gray reverse.

#### Conidial size

2.0–3.0 μm diam. (mean ±standard deviation, 2.4 ±0.3 μm).

#### Cardinal temperatures

Minimum 10°C, optimum 30°C, maximum 34°C. No growth at 4°C or 37°C.

#### Physiology

Growth at a_w_ ≈1.00 (0% NaCl, 0% MgCl_2_); optimum at a_w_ = 0.97 to 0.92 (4%-8% NaCl, 4%-6% MgCl_2_); maximum at a_w_ = 0.78 (28% NaCl, 13% MgCl_2_).

#### Extracellular enzyme activities

β-glucosidase at 10% NaCl; esterase at 0% NaCl; urease at 0% to 10% NaCl.

#### Habitat

Soil and house dust.

#### Distribution

Subtropical and tropical climates (Egypt, Uruguay, Indonesia and Micronesia).

#### Human and animal pathogenicity

Unknown.

#### Typification

Uruguay (Montevideo), from house dust collected by Z. Torrano in Dec 2008, culture isolated by K. Mwange, holotype, designated here, herbarium CBS H-22006, consisting of a freeze-dried living but metabolically inactivated deposit of spores and mycelium from MY50G). Living ex-type strain: EXF-8739.

#### Cultures examined

See S1 Table.

#### Diagnostic characters

Growth positive on media without additional solutes, such as MYA or MEA, conidia 2.0–3.0 μm diam., halotolerance up to 28% NaCl, chaotolerance up to 13% MgCl_2_, growth observed at 34°C, no β-glucosidase activity without NaCl, no esterase activity at 10% NaCl, occurrence in (sub)tropical climates.

#### Note

Two isolates from the marine sponges *Haliclona simulans* (ITS, GenBank FJ755832) and *Gelliodes carnosa* (ITS, FJ770080) from tropical Hainan Island coastal water [72] may represent *W*. *tropicalis*. Their sequences are identical or nearly identical with the ITS sequence of ex-type strain EXF-8739. This species was designated *W*. *sebi* clade 4 by Nguyen et al. [27].

## Discussion

The discovery of *Wallemia*, named after Mr. Wallem, a fishery inspector who sent *Wallemia*-infected fish for examination to Johan-Olsen in 1885, dates back to the 18^th^ and 19^th^ centuries, when clipfish was an important part of commercial fishing activities in Norway [73]. Species of *Wallemia* are among the few osmophilic fungi that can contaminate salted and dried cod. The monotypic genus included only *W*. *ichthyophaga* Johan-Olsen 1887 until von Arx in 1970 recognized *Sporendonema sebi* as a species of *Wallemia* [1], which provided the epithet for what today is the most frequently cited *Wallemia* species name.

From previous analyses of ITS sequences, it was suspected that *W*. *sebi* represents a genetically heterogeneous group consisting of at least two phylogenetically delineated clades [3]. In the companion study [27], genealogical concordance phylogenetic species recognition [67] based on sequences of the protein-encoding genes *rpb*2, *rpb*1, *MCM*7 and *tsr*1, clearly resolved the WSSC into four, statistically well-supported groups (clades 1–4). This conclusion was reaffirmed in this study by sampling strains from more diverse ecological niches. The approximately 350-bp partial sequence of *HAL*2 is used here for the first time as a phylogenetic marker gene. *HAL*2 contains a comparably high number of informative characters per site, is a single-copy gene [70], and easily amplifiable. Accordingly, *HAL*2 allows DNA barcode species identifications in *Wallemia*. Our phylogenetic analysis with *HAL*2, however, differed in some inferences in comparison to *rpb*2, *rpb*1, *MCM*7 and *tsr*1.

Our phylogenetic analyses infer that clade 1 members form a monophyletic and statistically supported group. Because this clade includes the ex-neotype strain of *W*. *sebi* CBS 818.96, it represents *W*. *sebi sensu stricto*. Strains of clade 1 typically originate from sea salt, hypersaline water, and air and dust in indoor environments from locations worldwide. It was also isolated from fresh water, seeds and other substrates with normal a_w_. *Wallemia sebi* can be disseminated through air and can colonize various low a_w_ substrates.

Our molecular data suggest that clade 2, described above as *W*. *mellicola*, is closely related to *W*. *sebi*. Clade 2 representatives are clearly resolved as a strongly supported monophyletic group on the basis of all tested loci but ITS. Because of its relatively large conidia (2.5–3.0 μm diam.), *W*. *mellicola* is morphologically distinguishable from *W*. *sebi*, which has conidia that are 1.5–2.5 μm diam. Physiologically, *W*. *sebi* differs from *W*. *mellicola* by its degree of halotolerance and chaotolerance. Its growth characters on MEA or MYA (5–7 mm diameter after 2 weeks; growth rate, 0.36 mm d^-1^), and the cerebriform colony structure on MYA distinguish *W*. *mellicola* from other *Wallemia* species. *Wallemia mellicola* was isolated from hypersaline water of solar salterns, salty, sugary and dried food products, and air, dust and surfaces in indoor environments worldwide and occasionally also from soil, forest plants, seeds, straw and pollen.

*Wallemia canadensis*, formerly referred to as *W*. *sebi* clade 3 [16,27], is best distinguished by its lower degree of halotolerance (0%-24% NaCl) and chaotolerance (0%-11% MgCl_2_), its cardinal growth temperatures, and its unique set of secondary metabolites. *Wallemia canadensis* is the only species of the WSSC that does not grow at 34°C. It has an optimal growth temperature at 24°C whereas the other species in the WSSC have an optimum growth temperature at 30°C. Strains of *W*. *canadensis* were isolated from house dust and soil in temperate and cold climates of North America (Canada).

*Wallemia tropicalis* is represented by only a few strains in clade 4, and was isolated from house dust and soil in (sub)tropical climates of South America, North Africa, South Asia, and Oceania. Its high degree of halotolerance (0%-28% NaCl) may explain why it could be isolated from marine sponges [72]. *Wallemia tropicalis* resembles *W*. *sebi*, but can grow at salinities of up to 28% NaCl *in vitro*, and represents the most halophilic species of the WSSC. It is therefore possible that the two isolates from marine sponges also belong to *W*. *tropicalis*. *Wallemia tropicalis* is also characterized by its particularly slow-growing colonies on media that lack additional solutes, such as MEA or MYA.

Secondary metabolite profiles generated provide meaningful support for the taxonomic structure suggested in our study (see also [74]). The secondary metabolites profiles of *W*. *sebi* and *W*. *mellicola* are hardly distinguishable, and most of the identified or separated compounds are formed by strains of both species. Inventories so far performed clearly indicate that these two species have similar habitats and they both occur relatively frequently in various environments worldwide. Probably they can also co-inhabit similar habitats. Our phylogenetic analyses support this interpretation, because the close phylogenetic relationship of *W*. *sebi* and *W*. *mellicola* is highly supported. Similarly, the unique secondary metabolite profile of *W*. *canadensis* supports its status as a distinct species, and none of its secondary metabolites were encountered in *W*. *sebi* or *W*. *mellicola*. It is possible that *W*. *canadensis* occupies different ecological niches in nature where other secondary metabolites are required or of advantage. The same may apply to *W*. *tropicalis*, for which no secondary metabolites were detected with the methods applied here.

With the exception of a few cases, the species of the WSSC are not associated with humans or animals. The few available reports suggest that they may act as opportunistic human pathogens, unless *W*. *sebi* strain CBS 196.56, from human skin [3], and *W*. *mellicola* strain EXF-8754, isolated from a subcutaneous lesion [69], present accidental airborne contaminants, which also is possible. There is one report of *W*. *sebi* as a member of bovine rumen microbiota (GenBank number JX240410), and one of *W*. *mellicola* from dog intestine (GenBank number EU486095 [50]).

A few nomenclatural issues concerning the name *W*. *muriae*, representatives of which were used as an outgroup in our phylogenetic analyses, warrant comment. The putative synonym *Hemispora stellata* Vuill. originated in 1906, and the epithet *muriae* in 1867 as a variety (*Torula epizoa* var. *muriae* J.J.Kickx). Names and epithets do not have priority outside of their rank (Art. 11.2, ICN, [66]), and at first glance *stellata* would have priority over *muriae*. However, the epithet *muriae* was raised to species rank in 1902 by Vestergren as *Torula muriae* (J.J.Kickx) Vesterg [75]. Accordingly, *W*. *muriae* (J.J.Kickx) Zalar & de Hoog [3] is still the nomenclaturally correct name for this fungus. The identity of *Oidium morrhuae* Farlow 1886, included as a tentative synonym of *W*. *muriae* by Zalar et al. [3] is unknown, but its description from a fish suggests that it is more likely to be *W*. *ichthyophaga*. The type of *O*. *morrhuae* has not been re-examined by a modern author. Because of the traction that the name *W*. *muriae* has attained in the literature (about 30 citations in Google Scholar, and 280 nucleotide sequence accessions in GenBank, as determined in Feb. 2015), we recommend its eventual inclusion on the lists of protected fungal names being prepared by nomenclatural specialists (Art. 14.13, ICN).

In the present study, we described species that originally were recognized on the basis of multi-locus sequence analyses [27] with phenotypic characters that included micromorphology and macromorphology, xerotolerance, halotolerance and chaotolerance, cardinal growth temperatures, and profiles of extracellular enzyme activities and secondary metabolites profiles. Here, we complete the formal taxonomic process of describing new species within the WSSC and provide a key to all of the proposed *Wallemia* species, which allows their identification on the basis of physiological, micromorphological and culture characters.

### Dichotomous key to *Wallemia* species

The micromorphological characters used in the key are from cultures grown on MY50G (MYA with addition of 50% sucrose) for 14 d at 24°C and 34°C; culture phenotypes and physiological characters are from colonies grown on MEA or MYA with the addition of sucrose (50%), NaCl (28%) and MgCl_2_ (15% and 17%) for 14 d at 24°C. For molecular barcode based identifications we suggest the generation of at least one protein-encoding marker in addition to the official fungal barcode ITS. Of the five loci tested here and in a companion study [27], *TSR*1 and *HAL*2 allow best DNA barcode based species identifications for the taxa of the WSSC.

1A —Colonies growing only on MYA or MEA with additional solutes (NaCl, glucose): conidia 2.5 μm to 5.0 μm diam.– 2

1B —Colonies growing on MYA or MEA without additional solutes: conidia 1.5 μm to 3.0 μm diam.– 3

2A —Colonies growing on MY50G: dark brown, with a cerebriform surface; conidia 3.5 μm to 5.0 μm diam.—*W*. *ichthyophaga*

2B —Colonies growing on MY50G: walnut brown, with a powdery surface; conidia 2.5 μm to 3.0 μm diam.—*W*. *muriae*

diam.diam.3A —Colonies growing on MEA or MYA plus 28% NaCl—5

3B —No growth on MEA or MYA plus 28% NaCl—6

4A —Colonies growing on MEA or MYA plus 15% to 17% MgCl_2_—*W*. *sebi*

4B —No growth on MEA or MYA plus 15% to 17% MgCl_2_—*W*. *tropicalis*

5A —Colonies growing at 34°C and on MEA or MYA 13% MgCl_2_—*W*. *mellicola*

5B —No growth at 34°C and on MEA or MYA plus 13% MgCl_2_—*W*. *canadensis*

## Supporting Information

S1 Fig Majority rule consensus tree of Bayesian MCMC sampling inferred from newly generated and GenBank accessed ITS sequences. Bayesian posterior probabilities are displayed at the nodes of the tree. The tree was rooted to the sequence of *W*. *muriae* ex-type strain CBS 116628 (AY302534). Labels provide information on strain numbers, origin and strain status. Red T, ex-type strains; red NT, ex-neotype strain; bold, strains included in physiological and morphological studies, and extracellular enzyme activities; underlined, strains included in studies of secondary metabolites.
(TIF)

S2 Fig Comparison of pair-wise distance (p-distance), alignment length, and parsimony informative characters of *HAL*2. The grey bars show the distribution range of the p-distance within clades while the blue bars show the range of p-distances between clades. The mean p-distances and the number of observations (N) used to calculate each mean are shown. AL = alignment length in base pairs. PIC = number and percentage of parsimony informative characters in the alignment.
(TIF)

S3 Fig Principal component analysis based on the production of secondary metabolites of the groups of strains from clades 1–3. No compounds were detected for clade 4 members grown on YES agar and CYAS.
(TIF)

S1 Table Strains included in the present study, with their original sources and GenBank accession numbers for ITS, *MCM*7, *TSR*1, *RPB*1, *RPB*2 and *HAL*2 sequences. Sequences for *MCM*7, *TSR*1, *RPB*1 and *RPB*2 from strain CBS 633.66 were extracted from the Joint Genome Institute (JGI) MycoCosm site.
(XLSX)

S2 Table Primer names and sequences. (XLSX)

S3 Table Alignment properties, selected nucleotide substitution models, Bayesian analysis settings, and the Bayesian posterior probabilities for clades 1–4. (XLSX)

S4 Table Growth parameters of *W*. *sebi*, *W*. *mellicola*, *W*. *tropicalis*, and *W*. *canadensis* on MYA with different water activities. (XLSX)

S5 Table Secondary metabolite patterns detected by HPLC analysis during the growth of *W*. *sebi*, *W*. *mellicola*, *W*. *tropicalis*, and *W*. *canadensis* on YES agar and CYAS. (XLSX)

S1 File Trees resulting from single gene phylogenetic analyses. (PDF)

## References

[1] von Arx JA. The genera of fungi sporulating in pure culture. Lehre: Cramer Verlag; 1970.

[2] Moore RT. The Dolipore/Parenthesome Septum in Modern Taxonomy In: Sneh B, Jabaji-Hare S, Neate S, Dijst G, editors. *Rhizoctonia* species: taxonomy, molecular biology, ecology, pathology and disease control. Dordrecht: Kluwer Academic Publisher; 1996 p. 13–35.

[3] Zalar P, de Hoog GS, Schroers HJ, Frank JM, Gunde-Cimerman N. Taxonomy and phylogeny of the xerophilic genus *Wallemia* (Wallemiomycetes and Wallemiales, cl. et ord. nov.). Antonie Van Leeuwenhoek. 2005;87:311–328. 15928984 10.1007/s10482-004-6783-x

[4] Nguyen HDT, Nickerson NL, Seifert KA. *Basidioascus* and *Geminibasidium*: a new lineage of heat-resistant and xerotolerant basidiomycetes. Mycologia. 2013;105:1231–1250. 10.3852/12-351 23709525

[5] Pitt JI, Hocking AD. Fungi and Food Spoilage. 2. ed London: Blackie Academic & Professional; 1997.

[6] Takahashi T. Airborne fungal colony-forming units in outdoor and indoor environments in Yokohama, Japan. Mycopathologia. 1997;139:23–33. 9511234 10.1023/a:1006831111595

[7] Zeng QY, Westermark SO, Rasmuson-Lestander A, Wang XR. Detection and quantification of *Wallemia sebi* in aerosols by real-time PCR, conventional PCR, and cultivation. Appl Environ Microbiol. 2004;70:7295–7302. 15574929 10.1128/AEM.70.12.7295-7302.2004 PMC535157

[8] Fröhlich-Nowoisky J, Pickersgill DA, Després VR, Pöschl U. High diversity of fungi in air particulate matter. Proc Natl Acad Sci U S A. 2009;106:12814–12819. 10.1073/pnas.0811003106 19617562 PMC2722276

[9] Amend AS, Seifert KA, Samson R, Bruns TD. Indoor fungal composition is geographically patterned and more diverse in temperate zones than in the tropics. Proc Natl Acad Sci U S A. 2010;107:13748–13753. 10.1073/pnas.1000454107 20616017 PMC2922287

[10] Sakamoto T, Torii S, Yamada M, Urisu A, Iguchi H, Ueda M, et al Allergenic and antigenic activities of the osmophilic fungus *Wallemia sebi* asthmatic patients. Arerugi. 1989;38:352–359. 2783035

[11] Hanhela R, Louhelainen K, Pasanen A. Prevalence of microfungi in Finnish cow barns and some aspects of the occurrence of *Wallemia sebi* and Fusaria. Scand J Work Environ Health. 1995;21:223–228. 7481610 10.5271/sjweh.31

[12] Lappalainen S, Pasanen AL, Reiman M, Kalliokoski P. Serum IgG antibodies against *Wallemia sebi* and Fusarium species in Finnish farmers. Ann Allergy Asthma Immunol. 1998;81:585–592. 9892031 10.1016/S1081-1206(10)62710-X

[13] Reboux G, Piarroux R, Mauny F, Madroszyk A, Millon L, Bardonnet K, et al Role of molds in farmer’s lung disease in eastern France. Am J Respir Crit Care Med. 2001;163:1534–1539. 11401869 10.1164/ajrccm.163.7.2006077

[14] Roussel S, Reboux G, Dalphin JC, Laplante JJ, Piarroux R. Evaluation of salting as a hay preservative against farmer’s lung disease agents. Ann Agric Environ Med. 2005;12:217–221. 16457476

[15] Lappalainen MHJ, Hyvärinen A, Hirvonen MR, Rintala H, Roivainen J, Renz H, et al High indoor microbial levels are associated with reduced Th1 cytokine secretion capacity in infancy. Int Arch Allergy Immunol. 2012;159:194–203. 10.1159/000335596 22678428

[16] Desroches TC, McMullin DR, Miller JD. Extrolites of *Wallemia sebi*, a very common fungus in the built environment. Indoor Air. 2014;1–10. Available: http://www.ncbi.nlm.nih.gov/pubmed/24471934. 10.1111/ina.12081 24471934

[17] Miller JD, Sun M, Gilyan a, Roy J, Rand TG. Inflammation-associated gene transcription and expression in mouse lungs induced by low molecular weight compounds from fungi from the built environment. Chem Biol Interact. 2010;183:113–124. 10.1016/j.cbi.2009.09.023 19818335

[18] Frank M, Kingston E, Jeffery JC, Moss MO, Murray M, Simpson TJ, et al *Walleminol and Walleminone*, *novel caryophyllenes from the toxigenic fungus* Wallemia sebi. Tetrahedron Lett. 1999;40:133–136.

[19] Takahashi I, Maruta R, Ando K, Yoshida M, Iwasaki T, Kanazawa J, et al UCA1064-B, a new antitumor antibiotic isolated from *Wallemia sebi*: production, isolation and structural determination. J Antibiot. 1993;46:1312–1314. 8407596 10.7164/antibiotics.46.1312

[20] Moss MO. Recent studies of mycotoxins. J Appl Microbiol.1998;84:62S–76S. 10.1046/j.1365-2672.1998.0840s162s.x 9750363

[21] Wood GM, Mann PJ, Lewis DF, Reid WJ, Moss MO. *Studies on a toxic metabolite from the mould* Wallemia. Food Addit Contam. 1990;7:69–77. 2106458 10.1080/02652039009373822

[22] Peng XP, Wang Y, Liu PP, Hong K, Chen H, Yin X, et al *Aromatic compounds from the halotolerant fungal strain of* Wallemia sebi *PXP-89 in a hypersaline medium* . Archi Pharmacol Res. 2011;34:907–912. 10.1007/s12272-011-0607-0 21725811

[23] Botić T, Kralj Kunčič M, Sepčić K, Knez Z, Gunde-Cimerman N. Salt induces biosynthesis of hemolytically active compounds in the xerotolerant food-borne fungus *Wallemia sebi* . FEMS Microbiol Lett. 2012;326:40–46. 10.1111/j.1574-6968.2011.02428.x 22092533

[24] Kralj Kunčič M, Zajc J, Drobne D, Pipan Tkalec Ž, Gunde-Cimerman N. Morphological responses to high sugar concentrations differ from adaptation to high salt concentrations in the xerophilic fungi *Wallemia* spp. Fungal Biol. 2013;117:466–478. 10.1016/j.funbio.2013.04.003 23931114

[25] Kralj Kunčič M, Kogej T, Drobne D, Gunde-Cimerman N. *Morphological response of the halophilic fungal genus* Wallemia *to high salinity* . Appl Environ Microbiol. 2010;76:329–337. 10.1128/AEM.02318-09 19897760 PMC2798636

[26] Zajc J, Liu Y, Dai W, Yang Z, Hu J, Gostinčar C, et al Genome and transcriptome sequencing of the halophilic fungus *Wallemia ichthyophaga*: haloadaptations present and absent. BMC Genomics. 2013;14:617 Available: http://www.ncbi.nlm.nih.gov/pubmed/24034603. 10.1186/1471-2164-14-617 24034603 PMC3849046

[27] Nguyen HDT, Jančič S, Meijer M, Tanney JB, Zalar P, Gunde-Cimerman N, et al Application of the phylogenetic species concept to *Wallemia sebi* from house dust and indoor air revealed by multi-locus genealogical concordance. PLoS One. In press. 10.1371/journal.pone.0120894 PMC4370657 25799362

[28] Hallsworth JE, Yakimov MM, Golyshin PN, Gillion JL, D’Auria G, de Lima Alves F, et al Limits of life in MgCl_2_-containing environments: chaotropicity defines the window. Environ Microbiol. 2007;9:801–813. 17298378 10.1111/j.1462-2920.2006.01212.x

[29] Williams JP, Hallsworth JE. Limits of life in hostile environments: no barriers to biosphere function? Environ Microbiol. 2009;11: 3292–3308. 10.1111/j.1462-2920.2009.02079.x 19840102 PMC2810447

[30] Vandamme P, Pot B, Gillis M, de Vos P, Kersters K, Swings J. Polyphasic taxonomy, a consensus approach to bacterial systematics. Microbiol Rev. 1996;60:407–438. 8801440 10.1128/mr.60.2.407-438.1996 PMC239450

[31] Quaedvlieg W, Binder M, Groenewald JZ, Summerell BA, Carnegie AJ, Burgess TI, et al Introducing the Consolidated Species Concept to resolve species in the Teratosphaeriaceae. Persoonia. 2014;33:1–40. 10.3767/003158514X681981 25737591 PMC4312929

[32] Wilson EO. Consilience: the unity of knowledge. New York: Alfred A. Knopf, Inc.; 1998.

[33] Whewell W. The Philosophy of the inductive sciences, founded upon their history. 2 vols London: John W. Parker; 1840.

[34] Visagie CM, Hirooka Y, Tanney JB, Whitfield E, Mwange K, Meijer M, et al *Aspergillus*, *Penicillium* and *Talaromyces* isolated from house dust samples collected around the world. Stud Mycol. 2014;78:63–139. 10.1016/j.simyco.2014.07.002 25492981 PMC4255536

[35] White TJ, Bruns S, Lee S, Taylor J. Amplification and direct sequencing of fungal ribosomal RNA genes for phylogenetics In: Innis MA GD, Sninsky JJ, White TJ, editors. PCR Protocols: a Guide to Methods and Applications. San Diego: Academic Press; 1990 p. 315–322.

[36] Liu YJ, Whelen S, Hall BD. Phylogenetic relationships among ascomycetes: evidence from an RNA polymerase II subunit. Mol Biol Evol. 1999;16:1799–1808. 10605121 10.1093/oxfordjournals.molbev.a026092

[37] Hibbett DS. A phylogenetic overview of the Agaricomycotina. Mycologia. 2006;98:917–925. 17486968 10.3852/mycologia.98.6.917

[38] Matheny PB, Wang Z, Binder M, Curtis JM, Lim YW, Nilsson RH, et al Contributions of rpb2 and tef1 to the phylogeny of mushrooms and allies (Basidiomycota, Fungi). Mol Phylogenet Evol. 2007;43:430–451. 17081773 10.1016/j.ympev.2006.08.024

[39] Schmitt I, Crespo A, Divakar PK, Fankhauser JD, Herman-Sackett E, Kalb K, et al New primers for promising single-copy genes in fungal phylogenetics and systematics. Persoonia. 2009;23:35–40. 10.3767/003158509X470602 20198159 PMC2802727

[40] Vaupotič T, Gunde-Cimerman N, Plemenitaš A. Novel 3’-phosphoadenosine-5'-phosphatases from extremely halotolerant *Hortaea werneckii* reveal insight into molecular determinants of salt tolerance of black yeasts. Fungal Genet Biol. 2007;44:1109–1122. 17420146 10.1016/j.fgb.2007.02.005

[41] Katoh K, Kuma K, Toh H, Miyata T. MAFFT version 5: improvement in accuracy of multiple sequence alignment. Nucleic Acids Res. 2005;33:511–518. 15661851 10.1093/nar/gki198 PMC548345

[42] Gouy M, Guindon S, Gascuel O. SeaView version 4: A multiplatform graphical user interface for sequence alignment and phylogenetic tree building. Mol Biol Evol. 2010;27:221–224. 10.1093/molbev/msp259 19854763

[43] Swofford DL. PAUP* 4.0: phylogenetic analysis using parsimony (*and other methods). Sunderland, Massachusetts: Sinauer Associates; 2002.

[44] Darriba D, Taboada GL, Doallo R, Posada D. jModelTest 2: more models, new heuristics and parallel computing. Nat Methods. 2012;9:772 10.1038/nmeth.2109 22847109 PMC4594756

[45] Ronquist F, Teslenko M, van der Mark P, Ayres DL, Darling A, Höhna S, et al MrBayes 3.2: efficient Bayesian phylogenetic inference and model choice across a large model space. Syst Biol. 2012;61:539–542. 10.1093/sysbio/sys029 22357727 PMC3329765

[46] Cantrell SA, Tkavc R, Gunde-Cimerman N, Zalar P, Acevedo M, Báez-Félix C. Fungal communities of young and mature hypersaline microbial mats. Mycologia. 2013;105:827–836. 10.3852/12-288 23709488

[47] Yuan ZL, Zhang CL, Lin FC, Kubicek CP. Identity, diversity, and molecular phylogeny of the endophytic mycobiota in the roots of rare wild rice (*Oryza granulate*) from a nature reserve in Yunnan, China. Appl Environ Microbiol. 2010;76:1642–1652. 10.1128/AEM.01911-09 20038691 PMC2832373

[48] Hu L, Cao L, Zhang R. Bacterial and fungal taxon changes in soil microbial community composition induced by short-term biochar amendment in red oxidized loam soil. World J Microbiol Biotechnol. 2014;30:1085–1092. 10.1007/s11274-013-1528-5 24136343

[49] Rittenour WR, Ciaccio CE, Barnes CS, Kashon ML, Lemons AR, Beezhold DH, et al Internal transcribed spacer rRNA gene sequencing analysis of fungal diversity in Kansas City indoor environments. Environ Sci Process Impacts. 2014;16:33–43. 10.1039/c3em00441d 24258337 PMC3966654

[50] Suchodolski JS, Morris EK, Allenspach K, Jergens AE, Harmoinen JA, Westermarck E, et al Prevalence and identification of fungal DNA in the small intestine of healthy dogs and dogs with chronic enteropathies. Vet Microbiol. 2008;132:379–388. 10.1016/j.vetmic.2008.05.017 18586415

[51] Singh P, Raghukumar C, Verma P, Shouche Y. Fungal community analysis in the deep-sea sediments of the Central Indian Basin by culture-independent approach. Microb Ecol. 2011;61:507–517. 10.1007/s00248-010-9765-8 21057784

[52] Tamura K, Peterson D, Peterson N, Stecher G, Nei M, Kumar S. MEGA5: molecular evolutionary genetics analysis using maximum likelihood, evolutionary distance, and maximum parsimony methods. Mol Biol Evol. 2011;28:2731–2739. 10.1093/molbev/msr121 21546353 PMC3203626

[53] Lutzoni F, Kauff F, Cox CJ, McLaughlin D, Celio G, Dentinger B, et al Assembling the fungal tree of life: progress, classification, and evolution of subcellular traits. Am J Bot. 2004;91:1446–1480. 10.3732/ajb.91.10.1446 21652303

[54] Wheeler KA, Hocking AD, Pitt JI. Effects of temperature and water activity on germination and growth of *Wallemia sebi* . Trans Br Mycol Soc. 1988;90:365–368.

[55] Gams W, Hoekstra ES, Aptroot A. CBS Course of Mycology, 4th edn Baarn: Centraalbureau voor Schimmelcultures; 1998.

[56] Samson RA, Hoekstra ES, Frisvad JC, Filtenborg O. Introduction to food- and airborne fungi. Baarn: Centraalbureau voor Schimmelcultures; 2002.

[57] Paterson RRM, Bridge PD. Biochemical techniques for filamentous fungi. IMI Technical Habdbooks. Wallingford: CAB International; 1994.

[58] Strauss ML, Jolly NP, Lambrechts MG, van Rensburg P. Screening for the production of extracellular hydrolytic enzymes by non-*Saccharomyces* wine yeasts. J Appl Microbiol. 2001;91:182–190. 11442729 10.1046/j.1365-2672.2001.01379.x

[59] Brizzio S, Turchetti B, de García V, Libkind D, Buzzini P, van Broock M. Extracellular enzymatic activities of basidiomycetous yeasts isolated from glacial and subglacial waters of northwest Patagonia (Argentina). Can J Microbiol. 2007;53:519–525. 17612608 10.1139/W07-010

[60] de Garcia V, Zalar P, Brizzio S, Gunde-Cimerman N, van Broock M. *Cryptococcus species* (Tremellales) from glacial biomes in the southern (Patagonia) and northern (Svalbard) hemispheres. FEMS Microbiol Ecol. 2012;82:523–539. 10.1111/j.1574-6941.2012.01465.x 22861821

[61] Frisvad JC. Media and growth conditions for induction of secondary metabolite production In: Keller NP, Turner G, editors. Fungal Secondary Metabolism. Totowa, NJ: Humana Press; 2012 p. 47–58. 10.1007/978-1-62703-122-6_3 23065607

[62] Smedsgaard J. Micro-scale extraction procedure for standardized screening of fungal metabolite production in cultures. J Chromatogr A. 1997;760:264–270. 9062989 10.1016/s0021-9673(96)00803-5

[63] Frisvad JC, Thrane U. Standardized high-performance liquid chromatography of 182 mycotoxins and other fungal metabolites based on alkylphenone retention indices and UV-VIS spectra (diode array detection). J Chromatogr. 1987;404:195–214. 3680432 10.1016/s0021-9673(01)86850-3

[64] Sonjak S, Frisvad JC, Gunde-Cimerman N. Comparison of secondary metabolite production by *Penicillium crustosum* strains, isolated from Arctic and other various ecological niches. FEMS Microbiol Ecol. 2005;53:51–60. 16329929 10.1016/j.femsec.2004.10.014

[65] Rohlf FJ. NTSYS-pc: Numerical Taxonomy System, version 2.1 New York: Exeter Publishing. Ltd.; 2002.

[66] Mcneill J, Barrie FR, Buck WR, Demoulin V, Greuter W, Hawksworth DL, et al International Code of Nomenclature for algae, fungi, and plants (Melbourne Code). Königstein: Koeltz Scientific Books; 2012 10.11646/zootaxa.3852.5.10

[67] Taylor JW, Jacobson DJ, Kroken S, Kasuga T, Geiser DM, Hibbett DS, et al Phylogenetic species recognition and species concepts in fungi. Fungal Genet Biol. 2000;31: 21–32. 11118132 10.1006/fgbi.2000.1228

[68] Sampaio JP. Rhodotorula *Harrison (1928)* In: Kurtzman CP, Fell JW, Boekhout T, editors. The Yeasts, a taxonomic study. 5th edn Amsterdam: Elsevier; 2011 p 1873–1927.

[69] Guarro J, Gugnani HC, Sood N, Batra R, Mayayo E, Gené J, et al Subcutaneous phaeohyphomycosis caused by *Wallemia sebi* in an immunocompetent host. J Clin Microbiol. 2008;46:1129–1131. 10.1128/JCM.01920-07 18174296 PMC2268330

[70] Padamsee M, Kumar TK, Riley R, Binder M, Boyd A, Calvo AM, et al *The genome of the xerotolerant mold* Wallemia sebi *reveals adaptations to osmotic stress and suggests cryptic sexual reproduction* . Fungal Genet Biol. 2012;49:217–226. 10.1016/j.fgb.2012.01.007 22326418

[71] Gock MA, Hocking AD, Pitt JI, Poulos PG. Influence of temperature, water activity and pH on growth of some xerophilic fungi. Int J Food Microbiol. 2003;81:11–19. 12423914 10.1016/s0168-1605(02)00166-6

[72] Liu WC, Li CQ, Zhu P, Yang JL, Cheng KD. Phylogenetic diversity of culturable fungi associated with two marine sponges: *Haliclona simulans* and *Gelliodes carnosa*, collected from the Hainan Island coastal. Fungal Divers. 2010;42:1–15.

[73] Johan-Olsen O. Om sop på klipfisk den såkaldte mid. Christiania Videnskabs-Selskabs Forhandlinger; 1887.

[74] Frisvad JC, Andersen B, Thrane U. The use of secondary metabolite profiling in chemotaxonomy of filamentous fungi. Mycol Res. 2008;112:231–240. 10.1016/j.mycres.2007.08.018 18319145

[75] Vestergren T. Micromycetes Rariores Selecti, Fasc. XXII, No. 550. 1902.

