# Supplementary figures and images for: A Taxonomic Revision of the Wallemia sebi Species Complex
Source: PLoS One. 2015 May 27;10(5):e0125933. doi: 10.1371/journal.pone.0125933 (PMC4446336; doi:10.1371/journal.pone.0125933)

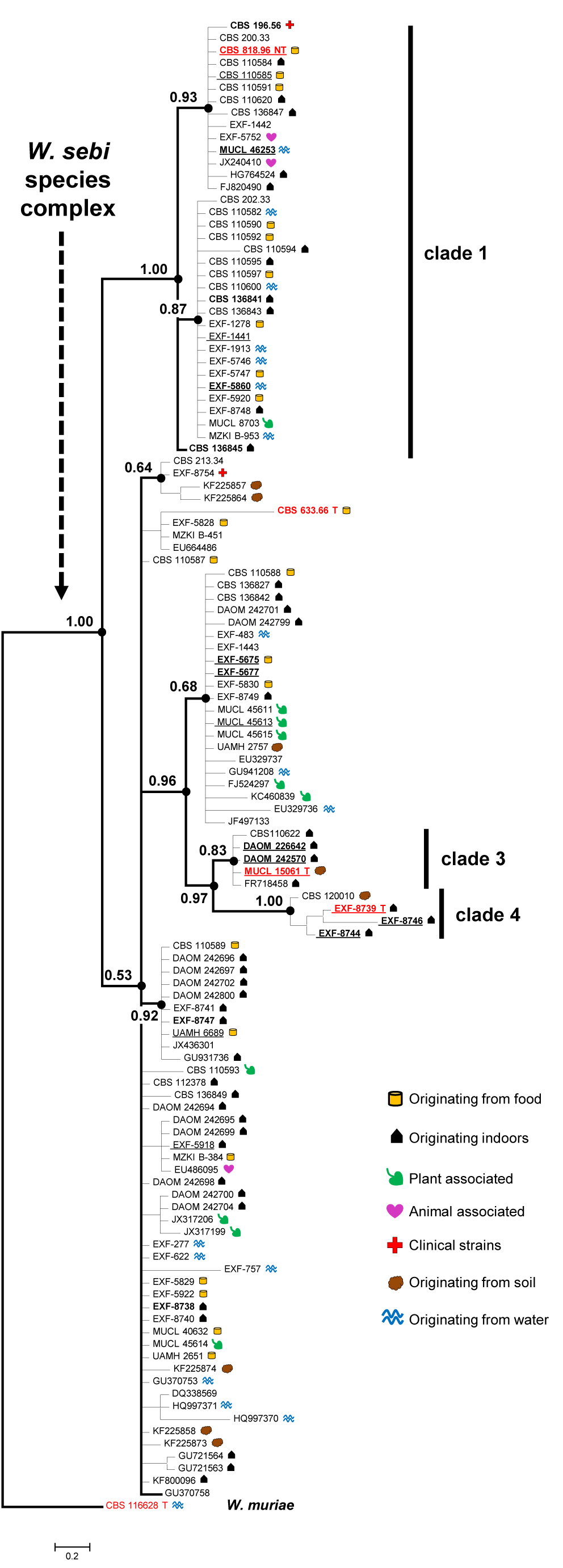

Supplement: S1 Fig — Bayesian posterior probabilities are displayed at the nodes of the tree. The tree was rooted to the sequence of W. muriae ex-type strain CBS 116628 (AY302534). Labels provide information on strain numbers, origin and strain status. Red T, ex-type strains; red NT, ex-neotype strain; bold, strains included in physiological and morphological studies, and extracellular enzyme activities; underlined, strains included in studies of secondary metabolites. (TIF) [file pone.0125933.s001.tif]

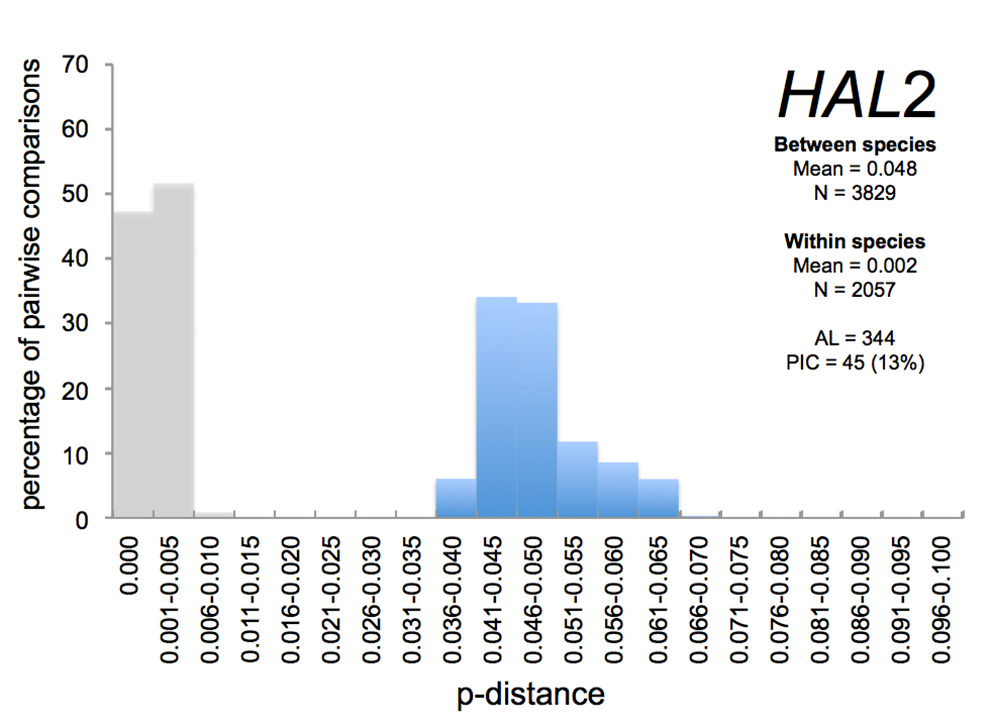

Supplement: S2 Fig — The grey bars show the distribution range of the p-distance within clades while the blue bars show the range of p-distances between clades. The mean p-distances and the number of observations (N) used to calculate each mean are shown. AL = alignment length in base pairs. PIC = number and percentage of parsimony informative characters in the alignment. (TIF) [file pone.0125933.s002.tif]

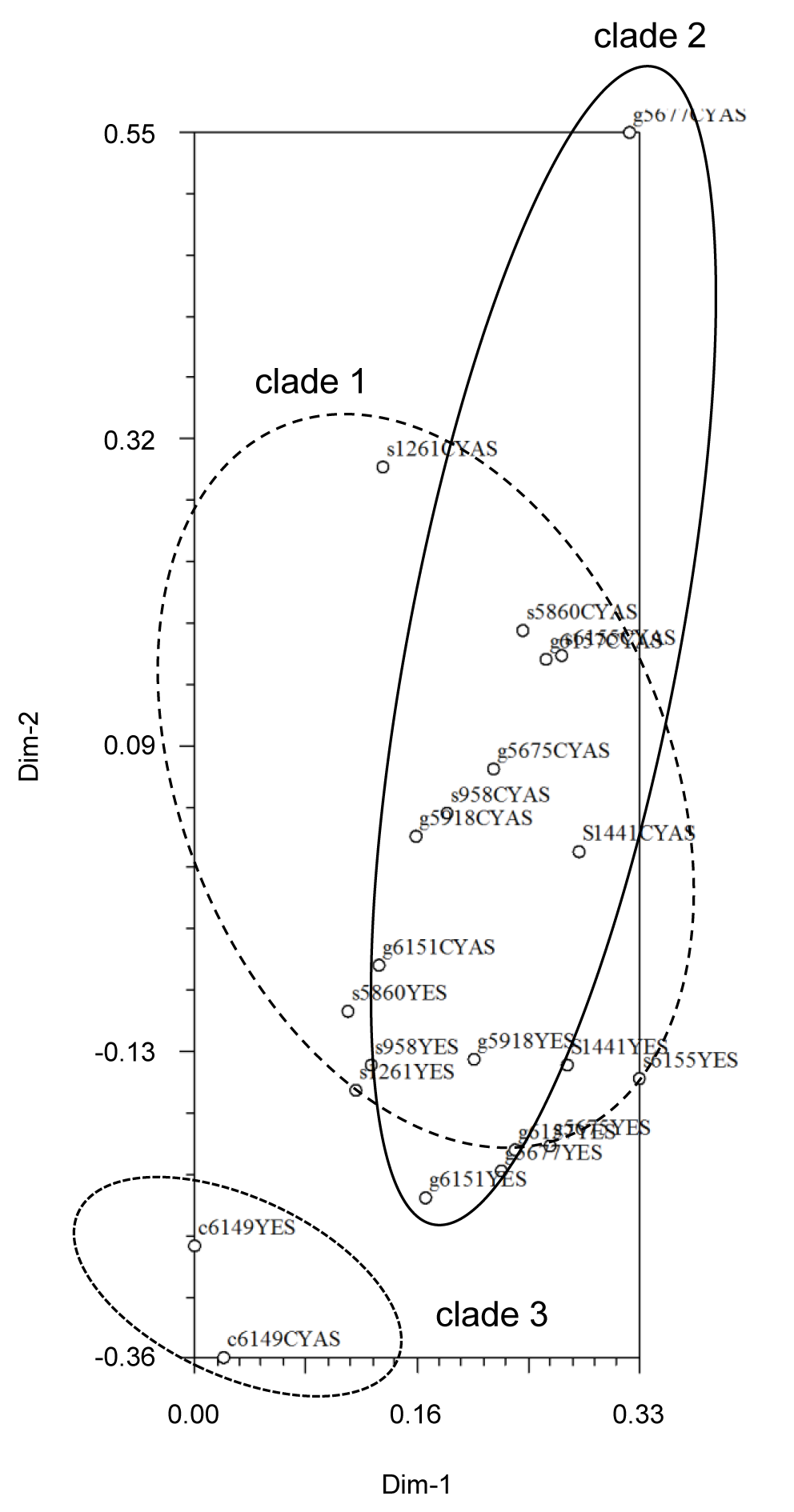

Supplement: S3 Fig — No compounds were detected for clade 4 members grown on YES agar and CYAS. (TIF) [file pone.0125933.s003.tif]

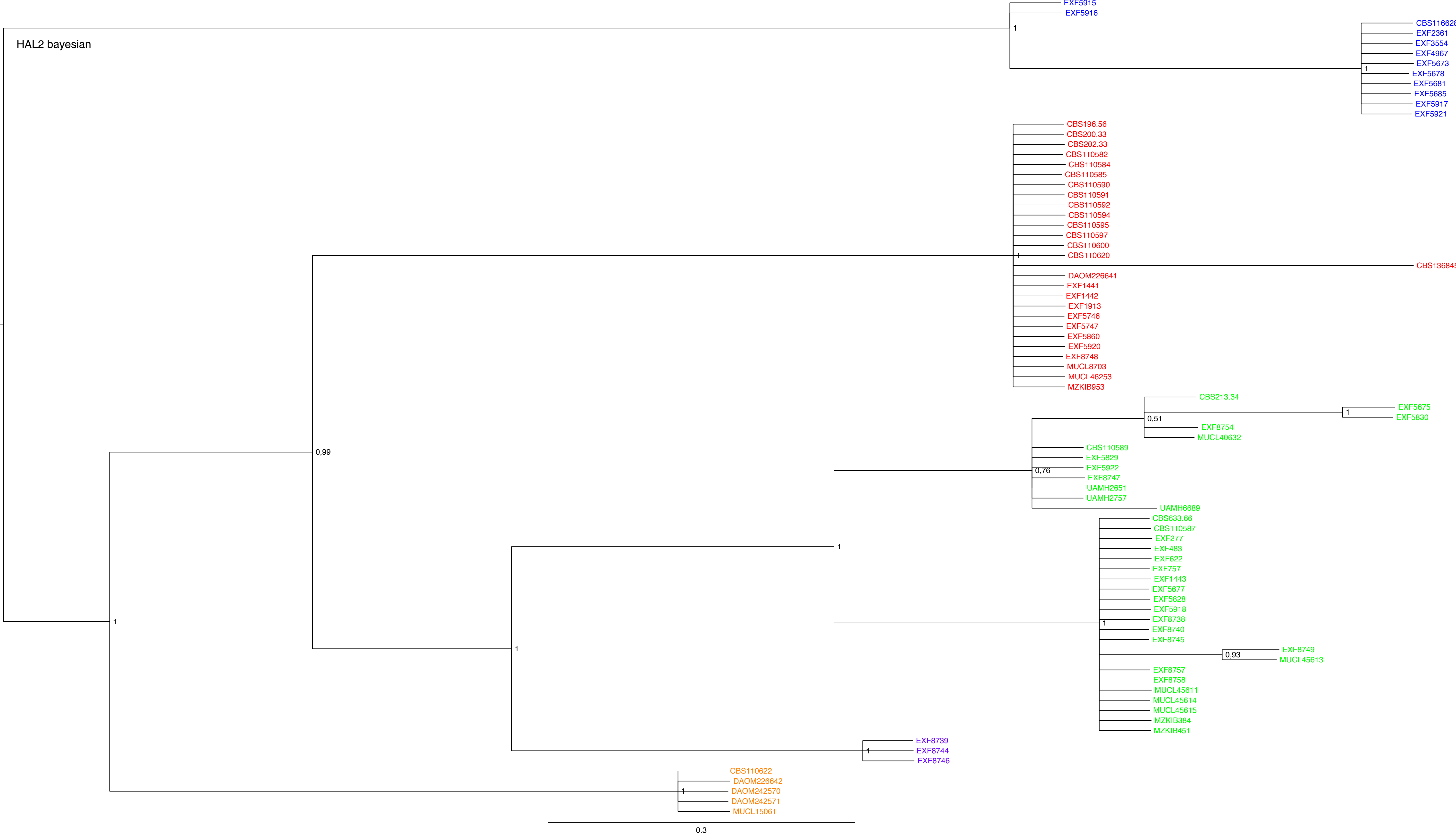

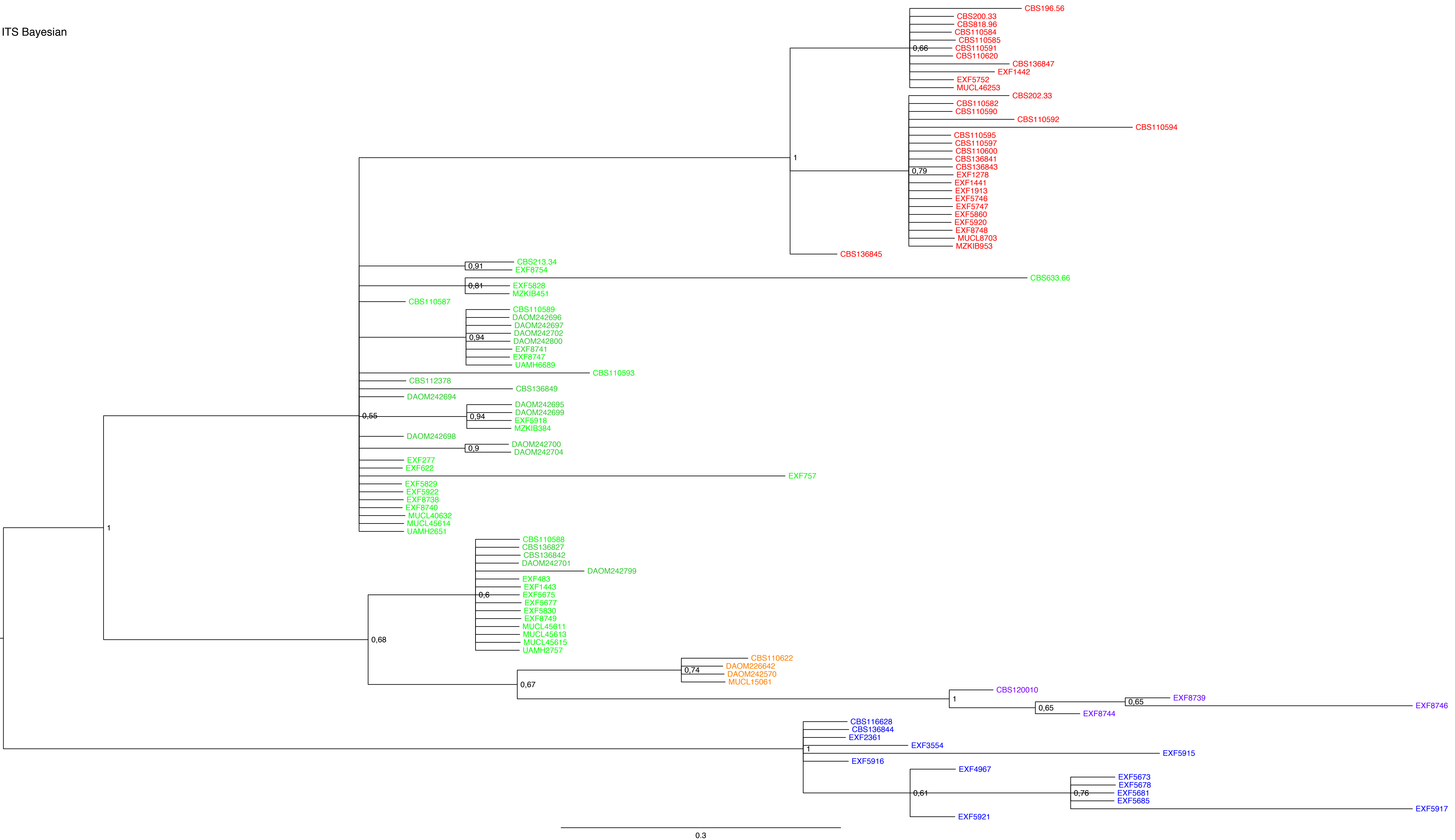

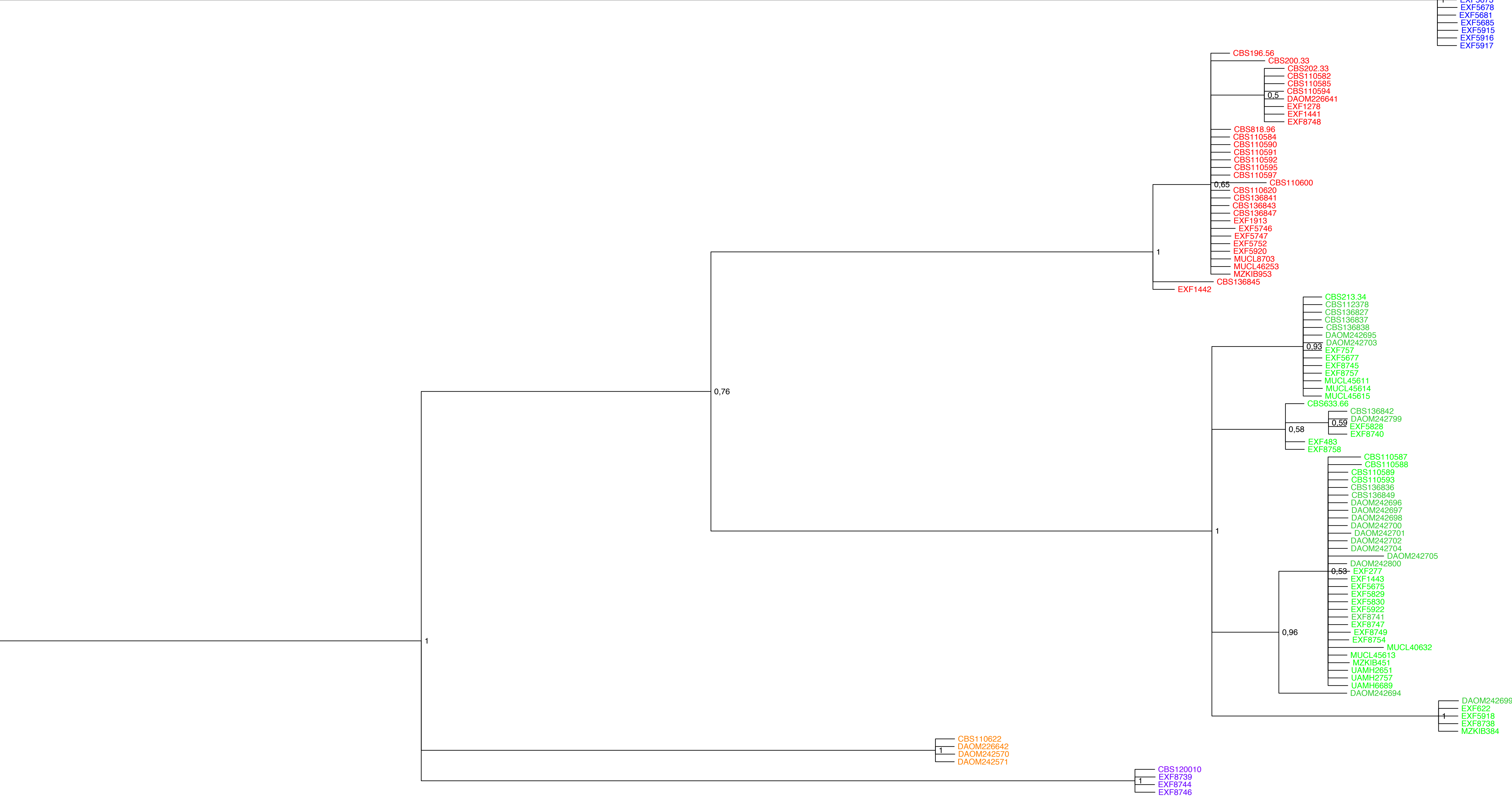

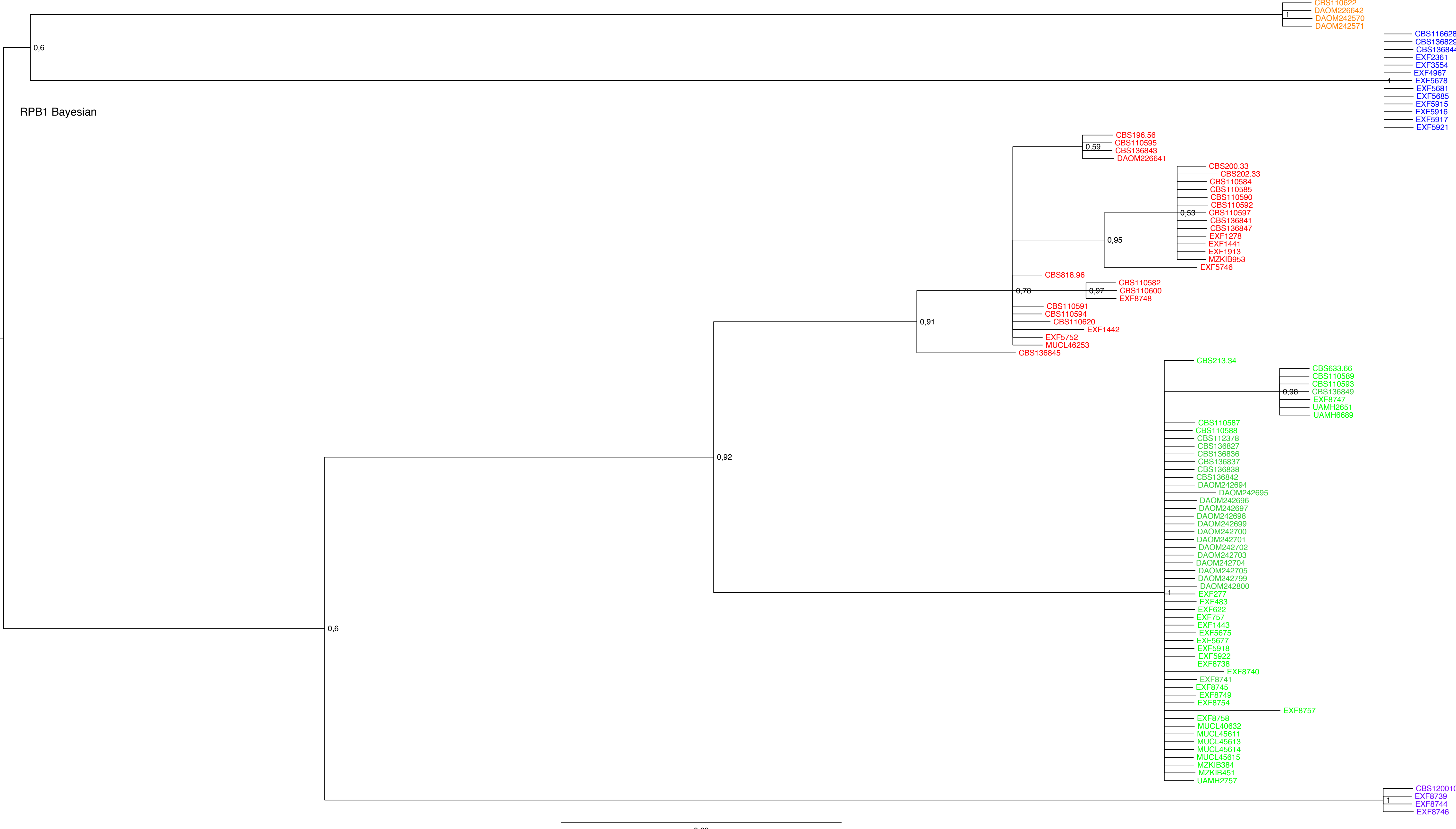

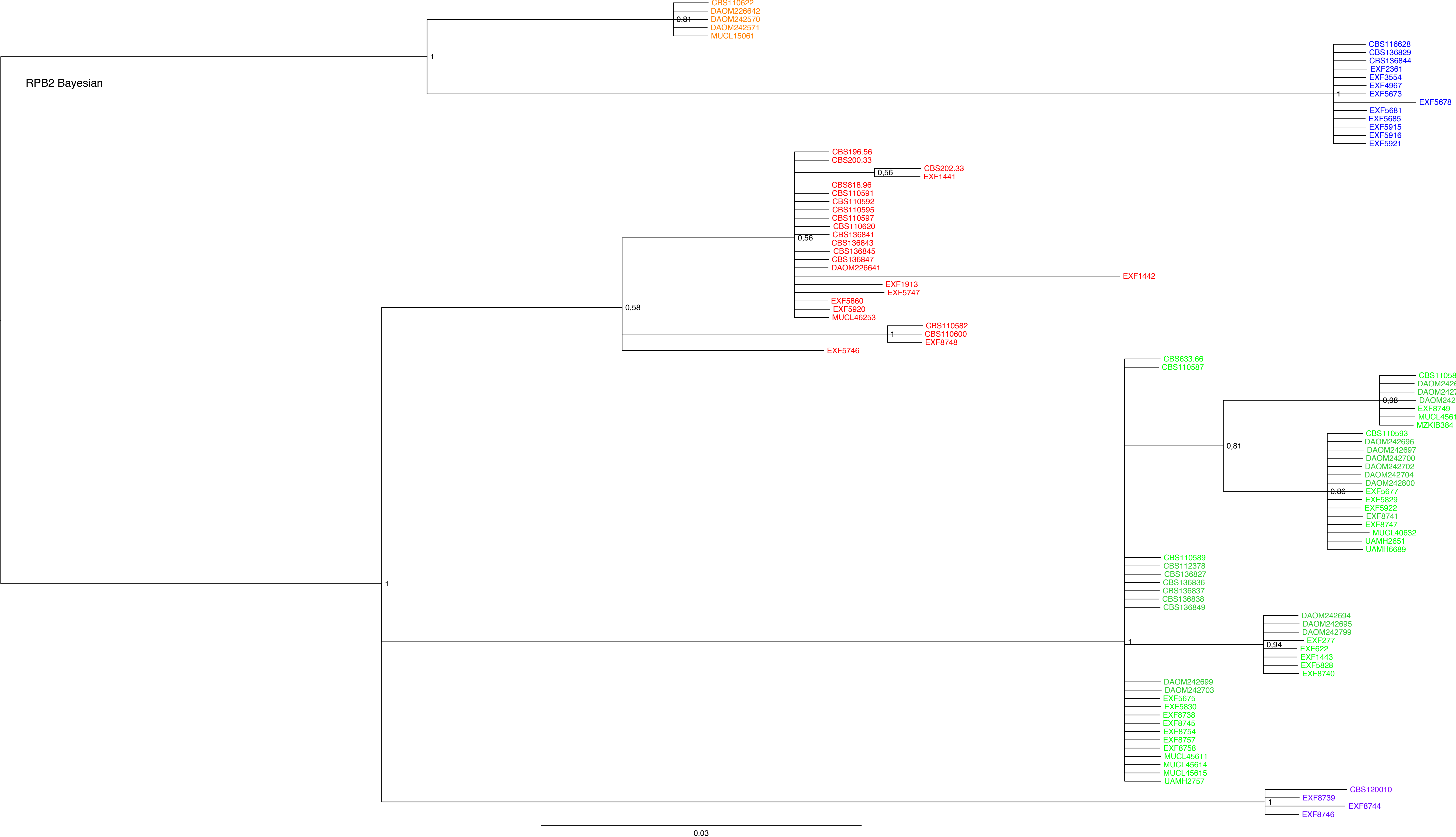

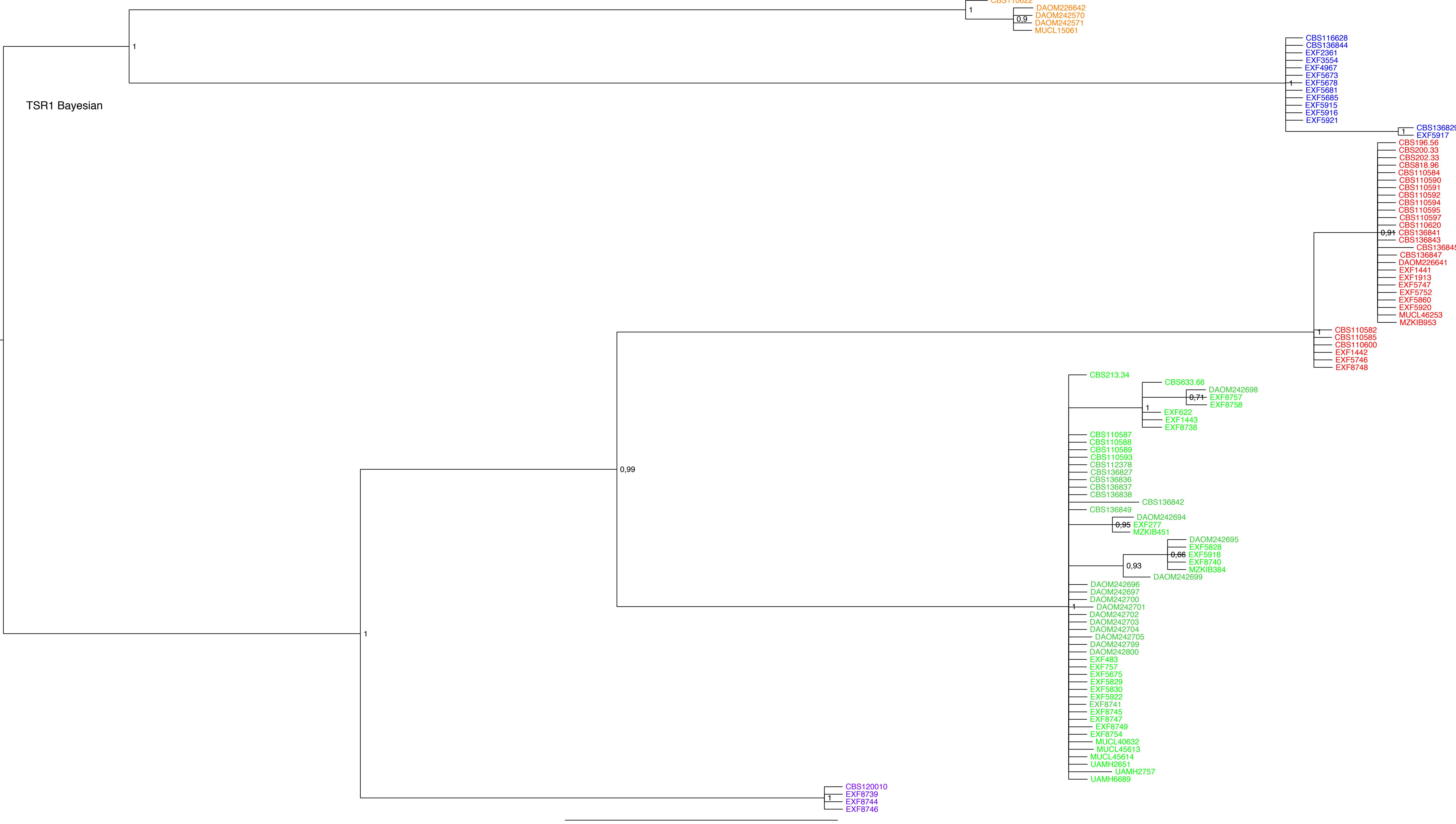

Supplement: S1 File — (PDF) [file pone.0125933.s009.pdf]
